# Room Temperature Reduction of Titanium Tetrachloride-Activated Nitriles to Primary Amines with Ammonia-Borane

**DOI:** 10.3390/molecules28010060

**Published:** 2022-12-21

**Authors:** P. Veeraraghavan Ramachandran, Abdulkhaliq A. Alawaed

**Affiliations:** Department of Chemistry, Purdue University, 560 Oval Drive, West Lafayette, IN 47907, USA

**Keywords:** reduction, ammonia-borane, nitrile, primary amines, titanium tetrachloride, catalysis

## Abstract

The reduction of a variety of aromatic and aliphatic nitriles, activated by a molar equivalent of titanium tetrachloride, has been achieved at room temperature using ammonia borane as a safe reductant. The corresponding methanamines were isolated in good to excellent yields following a simple acid-base workup.

## 1. Introduction

**Scheme 1:**
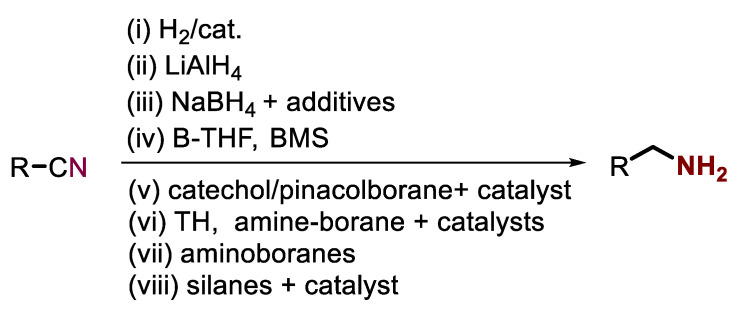
Reduction of organonitriles to primary amines.

The amine moiety in organic molecules is considered extremely important due to their multifaceted functions, especially in life sciences [1] and industrial chemistry [2,3]. Their applications encompass agro, materials, dye, textile, pharma, surfactant, plastic, and paper industries, to name a few. Accordingly, their syntheses have been the subject of intense research for organic chemists [4,5]. Primary amines function as intermediates or end-products in organic synthesis and have received their due attention. While reductive amination using ammonia or ammonium salts can be envisioned for their synthesis, it is often very challenging to perform [6,7,8]. Another simple process for primary amines is readily achieved via the reduction of organonitriles (Scheme 1) [9].

Catalytic hydrogenation (Scheme 1i) Of nitriles to amines depends on the reaction conditions [10] and often the intermediate imine-derivatives undergo side reactions to form secondary and/or tertiary amines. Among the hydride reducing agents, lithium aluminum hydride (LAH) can reduce nitriles to amines [11,12] (Scheme 1ii), whereas sodium borohydride (SBH) fails to achieve the reduction [13,14,15]. However, SBH with metal/metal salt additives [16,17,18,19,20], such as aluminum chloride, indium chloride [21], zinc chloride, R_2_SeBr_2_ [22], etc. has been utilized for this reduction (Scheme 1iii). Borane derivatives, such as borane-tetrahydrofuran (B-THF) and borane-dimethyl sulfide (BMS), have also been used for the reduction of nitriles [23,24] (Scheme 1iv). Oxoborane derivatives, such as catecholborane or pinacolborane, which are slow in their reactivity compared to borane or its alkyl derivatives have also been used to reduce nitriles by using transition metal activators as catalysts [25,26,27,28] (Scheme 1v). Several metal-catalyzed transfer hydrogenations (TH) using amine-boranes, including ammonia-borane to convert a nitrile to an amine have also been reported recently (Scheme 1vi) [29,30,31,32,33,34]. Diisopropylaminoborane generated via the dehydrogenation of the corresponding amine-borane has been shown to be effective for the reduction of nitriles to amines [35] (Scheme 1vii). The literature is also permeated with reports on the use of silane derivatives in the presence of activators for nitrile reduction [36,37,38,39,40,41,42] (Scheme 1viii).

**Scheme 2:**
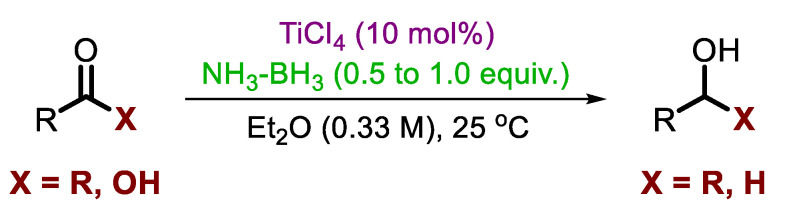
Reduction of carbonyls activated by TiCl_4_ using ammonia borane.

Most of these procedures have several serious drawbacks, such as air- and moisture-sensitivity of the reagents, expensive nature of the reagents or catalysts, the formation of dialkylamines, etc. Efficient procedures for the reduction of nitriles are still being sought actively. As part of our program on amine-boranes, we recently described the reduction of ketones [43] as well as carboxylic acids [44] using ammonia borane (AB, **1a**) in diethyl ether (Et_2_O), in the presence of sub-stoichiometric titanium tetrachloride as an activator (Scheme 2). While comparing the competitive reduction of a carboxylic acid and an organonitrile, under standard conditions, the latter was observed to be unreactive. Due to the importance of primary amines, we were eager to learn whether an organonitrile will succumb to the reaction under modified conditions. Accordingly, a systematic examination was initiated and reported herein is the facile conversion of nitriles to primary amines, at room temperature, with ammonia borane in the presence of a molar equivalent of titanium tetrachloride as the activator. Both aliphatic and aromatic nitriles underwent reduction, and the products were isolated, in good to excellent yields, using a simple acid-base workup.

## 2. Results and Discussion

The successful hydroboration of alkenes [45] and alkynes [46] with **1a** in refluxing tetrahydrofuran (THF), as opposed to the lack of any reaction at room temperature [47], led us to attempt the hydroboration (reduction) of a representative nitrile, benzonitrile (**2a**) with **1a** in refluxing THF. Surprisingly, even after 20 h, the reaction was only ~24% complete with one equiv. and ~60% complete with two equiv. of the reducing agent **1a** (entries 2 and 3, Table 1). This prompted a logical modification of the reaction of **2a**, which was conducted, under catalyzed conditions, in diethyl ether at room temperature. Unlike the sub-stoichiometric (10%) catalyst loading reported for the reduction of ketones and acids [43,44], the reaction was now performed in the presence of increasing amounts of the activator titanium tetrachloride as well as differing stoichiometries of the reducing agent, **1a**. The reaction was followed by thin layer chromatography for the disappearance of **2a**. After several attempts, we were delighted to observe that using a molar ratio of 1:2:0.7 for **2a**:**1a**: activator led to complete conversion of 2a to benzylamine (3a) in 77% isolated yield within 3 h (entry 4, Table 1). No conversion of benzonitrile was observed in the absence of the catalyst, confirming the crucial necessity of TiCl_4_ for this reduction (entry 1, Table 1). Remarkably, increasing the stoichiometry of TiCl_4_ to 1 equiv. and of **1a** to 2 equiv. provided an increase in yield (95%), while decreasing the reaction time to an hour (entry 5, Table 1). Decreasing the reagent load of 1a to 1.5 equiv., however, resulted in an inefficient reaction and the yield decreased to 71% (entry 6, Table 1). Increasing the reaction time up to 24 h did not have any effect on the yield.

The conversions and reaction rates for amine formation from nitriles strongly depended on the reaction parameters, such as solvent, Lewis acid and its equivalences, as well as the amine-borane used. The effect of the solvent was exemplified by replacing Et_2_O, with dichloromethane (CH_2_Cl_2_), THF, and pentane under similar conditions. These observations confirmed that Et_2_O is the best solvent to effect the transformation effortlessly (entries 7–9 in Table 1).

Next, other common Lewis acids, such as TiBr_4_, HfCl_4_, BF_3_•Et_2_O, AlCl_3_, and FeCl_3_, were examined (entries 10–14, Table 1). Among these catalysts, TiBr_4_ showed good catalytic activity (entry 10). However, due to the relatively higher cost of TiBr_4_, TiCl_4_ was used as the catalyst for subsequent studies.

**Figure 1:**
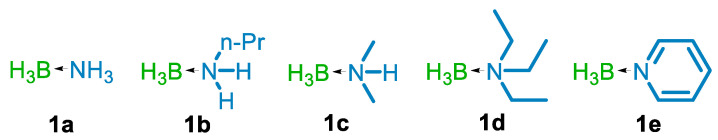
Amine-boranes examined for the reduction of organonitriles.

The effect of the amine-borane reductant was then examined by incorporating amine-boranes of differing substitutions on the nitrogen, prepared in our laboratory [48,49], in place of **1a**. Thus, 1°-(*n*-propylamine-borane, **1b**) 2°-(dimethylamine-borane, **1c**), 3°-(triethylamine-borane, **1d**) and heteroaromatic (pyridine-borane, **1e**) were examined (Figure 1) and the results reveal that **1a** is the most efficient among all the amine-boranes tested (entries 15–18 in Table 1). Notably, when triethylamine-borane (**1d**) was used, no reduction was observed (entry 17, Table 1).

**Figure 2:**
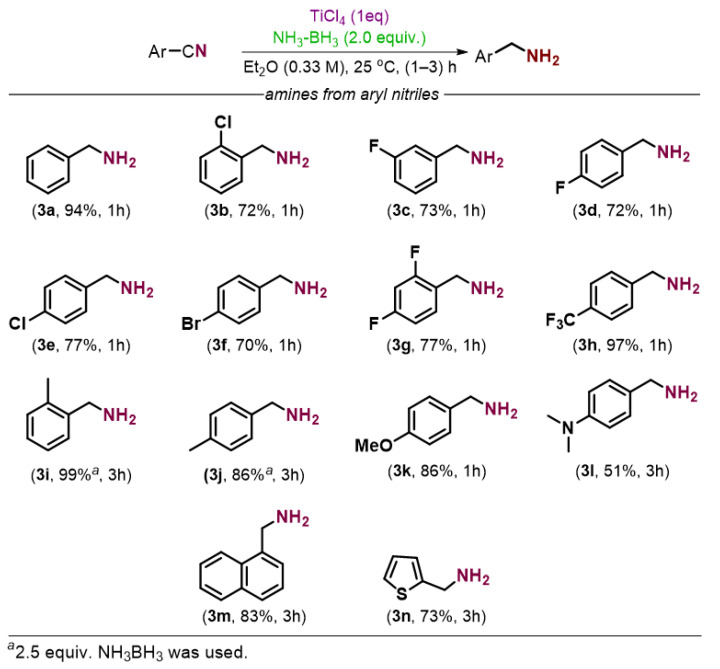
Reduction of activated aromatic nitriles with ammonia borane.

Having optimized the reaction conditions to achieve the reduction of benzonitrile in 95% yield, the scope of the methodology was studied with respect to the organonitrile partner (Figure 2). Initially, the effect of substitutions on the benzene ring at the *ortho-*, *meta*-, and *para*-positions was evaluated. Thus, *ortho*-chlorobenzonitrile (**2b**), *meta*-fluorobenzonitrile (**2c**), and *para*-fluoro-(**2d**), -chloro-(**2e**), and -bromo-(**2f**) benzonitriles *were converted to* the corresponding amines (**3b**–**3f**) in 70–72% yields, respectively. Additionally, 2,4-difluorobenzonitrile (**2g**) provided the desired benzylamine product **3g** in 77% yield. No dehalogenation product was observed in all these cases.

Reductions of benzonitrile with an electron-deficient group on the aromatic ring, for example, 4-trifluoromethylbenzonitrile (**2h**) underwent the reduction efficiently to the corresponding amine **3h** in almost quantitative yield 97%, indicating that weak electron-withdrawing groups have no impact on this transformation. The electron-donating 2- and 4- methyl (**2i**–**2j**) did not inhibit the formation of amines (**3i**–**3j**), isolated in 99% and 86% yields in 3 h, respectively, although a slightly higher molar equivalent of **1a** (2.5 equiv.) was necessary for complete reduction. Furthermore, the reaction of increased electron-donating 4-methoxybenzonitrile was converted to the desired product methanamine **3k** in good yield (86%). It should be noted that higher temperatures were required when diisopropylaminoborane reagent was used for reduction of **2k** [35]. However, *para*-*N*, *N*-dimethylaminobenzonitrile (**2l**) provided the corresponding aminobenzylamine (**3l**), *albeit* in diminished yield (51%) even when the reaction was extended to 24 h. The sluggish reactivity was attributed to the deleterious effect of the dimethylamino group, which might be exchanging borane with ammonia and rendering the reduction ineffective or due to the deactivation of the catalyst by complexation with the non-bonding electrons on nitrogen.

As a representative of a bulky aryl nitrile, 1-cyanonaphthalene (**2m**) was subjected to the new ammonia borane reduction under the optimized conditions when the corresponding 1-naphthylmethanamine (**3m**) was isolated in 83% yield. In addition, a representative heteroaromatic nitrile, 1-thiophenenitrile (**2n**) also proved highly amenable to the reaction conditions and afforded thiophenylmethanamine (**3n**) in 73% yield.

**Figure 3:**
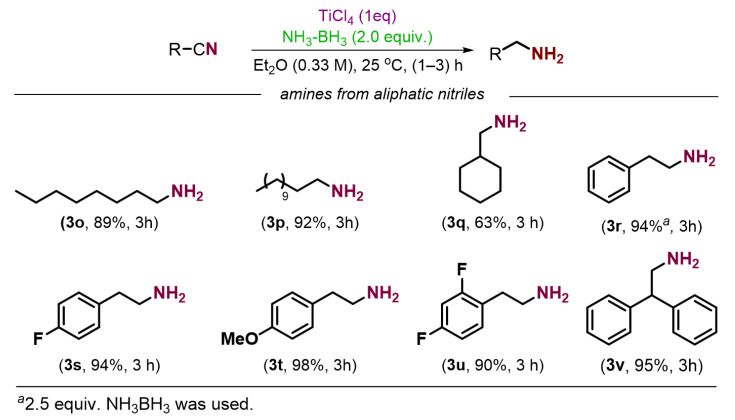
Reduction of activated aliphatic nitriles with ammonia borane.

Reduction of alkyl nitriles is considered a challenge and numerous methodologies have failed to reduce aliphatic nitriles to primary amines, mainly due to a competitive deprotonation of the acidic α-proton prior to the reduction of the nitrile moiety. Accordingly, a series of straight chain and branched aliphatic nitriles were also included in the study. We were pleased to observe that the ammonia-borane/TiCl_4_ reducing system is effective for the reduction of these nitriles as well (Figure 3). Our catalytic system does not induce deprotonation and, indeed, all the aliphatic nitriles were reduced, within 3 h, to their corresponding amines at room temperature in excellent yields. For example, acyclic octane- (**2o**) and dodecanenitrile (**2p**) were reduced to the amines **3o** and **3p**, respectively, in 89% and 92% yields. A branched nitrile, cyclohexanenitrile (**2q**) was also reduced, *albeit*, in a decreased 63% yield, to the corresponding methanamine **3q**. Additionally, substituted, and unsubstituted 2-phenylethanenitriles were examined and all of them provided the corresponding amines in >90% yields. Thus, the parent 2-phenylethanenitrile (**2r**), 2-(4-fluorophenyl)ethanenitrile (**2s**), 2-(4-methoxyphenyl)ethanenitrile (**2t**) and 2-(2′,4′-difluorophenyl)ethanenitrile (**2u**) with electron-neutral,-poor, and -rich substituents were converted to the corresponding amines **3r**–**3t** in 90%–98% yields. Gratifyingly, even a highly hindered ethanenitrile derivative, such as α,α-diphenylethanenitrile (**2v**), was reduced to the corresponding β,β-diphenylethylamine (**3v**) in quantitative yield 95%.

## 3. Materials and Methods

### 3.1. General Information

Ammonia-borane [50] and other amine-boranes used in this study were prepared according to our earlier published procedures [48,49]. Other reagents and solvents as well as the amines or amine-hydrochlorides to prepare the amine-boranes were purchased from Sigma-Aldrich or Oakwood chemicals. The nitriles, amines, sodium bicarbonate, and sodium borohydride were used as received. Anhydrous diethyl ether was prepared by distillation over sodium-benzophenone and anhydrous dichloromethane was prepared by distillation over calcium hydride and stored under nitrogen atmosphere. Thin layer chromatography (TLC) was performed on silica gel F60 plates and visualized under UV light or ceric ammonium molybdate solution. The structures of the product amines were confirmed by nuclear magnetic resonance (NMR) spectroscopy and measured in δ values in parts per million (ppm). ^1^H NMR spectra of reduction products were recorded on a Bruker 400 MHz spectrometer at ambient temperature and calibrated against the residual solvent peak of CDCl_3_ (ẟ = 7.26 ppm) as an internal standard. The ^13^C NMR spectra were reported at 101MHz (297 K) and calibrated using CDCl_3_ (ẟ = 77.0 ppm) as an internal standard. Coupling constants (*J*) are given in hertz (Hz), and signal multiplicities are described of NMR data as s = singlet, d = doublet, t = triplet, dd = doublet of doublets, dt = doublet of triplets, qd = quartet of doublets, q = quartet, quint and p = pentet, m = multiplet, and br = broad. ^11^B, ^1^H (300 MHz), and ^13^C NMR (75 MHz) spectra of synthesized amine-boranes were recorded at room temperature on a Varian INOVA or MERCURY 300 MHz NMR instrument. ^11^B NMR spectra were recorded at 96 MHz and chemical shifts were reported relative to the external standard, BF_3_:OEt_2_ (ẟ = 0 ppm).

### 3.2. Experimental

#### 3.2.1. General Procedure for the Preparation of Amines from Nitriles

The preparation of benzylamine from benzonitrile is typical. A 50 mL oven dried round bottom flask was charged with benzonitrile (1 mmol, 1 equiv.) and a magnetic stirring bar. The flask was sealed using a rubber septum. After purging the flask with nitrogen, dry diethyl ether (or other solvents) (3 mL) was added, and the solution was cooled to 0 °C with an ice bath. Subsequently, TiCl_4_ (or other Lewis acids) (1 mmol, 1 equiv.) was added to the solution, dropwise via syringe if a liquid or by temporarily removing the septum (under a flow of nitrogen) if a solid. The septum was then carefully opened (under a flow of nitrogen) and ammonia borane (or other solid amine-borane) (2.0 mmol, 2.0 equiv.) was added slowly to the reaction mixture (liquid amine-boranes were added via a syringe). Upon complete addition, the reaction flask was again sealed with a septum. After stirring at 0 °C for 1 min, the reaction mixture was allowed to warm to room temperature, stirred and monitored by TLC for completion (disappearance of the starting nitrile), when the crude mixture was brought to 0 °C using an ice bath and quenched by the slow addition of cold 3 *M* HCl. The acidic solution was stirred for 30 min, made basic with 3 M NaOH to pH 11, transferred to a separatory funnel and extracted with diethyl ether (2 × 15 mL). The combined organic layers were washed with brine (1 × 3 mL), dried over anhydrous sodium sulfate, filtered through cotton, and concentrated under aspirator vacuum using a rotary evaporator. Any remaining traces of solvent were removed by subjecting to high vacuum for 30 min. The product amines were characterized using ^1^H and ^13^C NMR spectroscopy and compared with those reported in the literature and their references have been included. The spectra are available in Supporting Information. The results from the optimization experiments are shown in Table 1. Ammonia borane as the reductant and titanium chloride as the Lewis acid in diethyl ether solvent was established as the optimal procedure for subsequent reactions.

#### 3.2.2. Characterization of Product Amines

*Benzylamine (**3a**)***;** The compound was prepared as described in the general procedure (colorless oil, yield = 100 mg, 94%); **^1^H NMR** (400 MHz, CDCl_3_) δ 7.35–7.30 (m, 4H), 7.27–7.23 (m, 1H), 3.87 (s, 2H), 1.51 (s, 2H). **^13^C NMR** (101 MHz, CDCl_3_) δ143.2, 128.4, 127.0, 126.7, 46.4. Compound characterization is in accordance with previous reports [51].

*2-Chlorobenzylamine (**3b**)*; The compound was prepared as described in the general procedure (colorless oil, yield = 102 mg, 72%); **^1^H NMR** (400 MHz, CDCl_3_) δ 7.37–7.30 (m, 2H), 7.27–7.13 (m, 3H), 3.90 (d, *J* = 2.5 Hz, 2H), 1.60 (s, 2H). **^13^C NMR** (101 MHz, CDCl_3_) δ140.5, 133.2, 129.4, 128.8, 128.1, 127.0, 44.5. Compound characterization is in accordance with previous reports [52].

*3-Flourobenzylamine (**3c**)*; The compound was prepared as described in the general procedure (colorless oil, yield = 91 mg, 73%); **^1^H NMR** (400 MHz, CDCl_3_) δ 7.33–7.23 (m, 1H), 7.05 (dd, *J* = 17.0, 8.4 Hz, 2H), 6.92 (t, *J* = 7.8 Hz, 1H), 3.86 (s, 2H), 1.48 (s, 2H). **^13^C NMR** (101 MHz, CDCl_3_) δ161.7, 145.8, 129.9, 129.8, 122.4, 113.9, 113.7, 113.6, 113.4, 45.9. **^19^F NMR** (376 MHz, CDCl_3_) δ −114.9. Compound characterization is in accordance with previous reports [53].

*4-Fluorobenzylamine (**3d**)***;** The compound was prepared as described in the general procedure (yellow oil, yield = 90 mg, 72%); **^1^H NMR** (400 MHz, CDCl_3_) δ 7.27 (dd, *J* = 8.5, 5.5 Hz, 2H), 7.01 (t, *J* = 8.7 Hz, 2H), 3.84 (s, 2H), 1.44 (s, 2H). ^13^C NMR (101 MHz, CDCl_3_) δ162.9, 160.5, 138.8, 128.6, 128.5, 115.3, 115.0, 45.7. **^19^F NMR** (376 MHz, CDCl_3_) δ −117.9. Compound characterization is in accordance with previous reports [53].

*4-Chlorobenzylamine (**3e**)*; The compound was prepared as described in the general procedure (colorless oil, yield = 109 mg, 77%); **^1^H NMR** (400 MHz, CDCl_3_) δ 7.29 (d, *J* = 7.7 Hz, 2H), 7.25 (d, *J* = 7.0 Hz, 2H), 3.84 (s, 2H), 1.53 (s, 2H). **^13^C NMR** (101 MHz, CDCl_3_) δ141.5, 132.4, 128.5, 128.4, 45.7. Compound characterization is in accordance with previous reports [51].

*4-Bromobenzylamine (**3f**)***;** The compound was prepared as described in the general procedure (colorless oil, yield = 130 mg, 70%); **^1^H NMR** (400 MHz, CDCl_3_) δ 7.42 (d, *J* = 8.0 Hz, 2H), 7.16 (d, *J* = 8.2 Hz, 2H), 3.79 (s, 1H), 1.53 (s, 1H). **^13^C NMR** (101 MHz, CDCl_3_) δ 142.0, 131.4, 128.7, 120.4, 45.7. Compound characterization is in accordance with previous reports [52].

*2,4-Diflourobenzylamine (**3g**)*; The compound was prepared as described in the general procedure (colorless oil, yield = 110 mg, 77%); **^1^H NMR** (400 MHz, CDCl_3_) δ 7.33–7.23 (m, 1H), 6.87–6.72 (m, 2H), 3.84 (s, 2H), 1.52 (s, 2H). **^13^C NMR** (101 MHz, CDCl_3_) δ163.2, 161.9, 159.6, 129.9, 129.8, 129.7, 126.1, 126.0, 111.1, 110.9, 110.8, 103.9, 103.7, 103.4, 39.95, 39.91. **^19^F NMR** (376 MHz, CDCl_3_) δ −113.9, −117.4.

*(4-(Trifluoromethyl)phenyl)methanamine (**3h**)***;** The compound was prepared as described in the general procedure (colorless oil, yield = 170 mg, 97%); **^1^H NMR** (400 MHz, CDCl_3_) δ 7.58 (d, *J* = 8.0 Hz, 2H), 7.42 (d, *J* = 8.1 Hz, 2H), 3.93 (s, 2H), 1.61 (s, 1H). **^13^C NMR** ^13^C NMR (101 MHz, CDCl_3_) δ146.9, 128.5, 127.2, 125.35, 125.31, 122.8, 45.8. **^19^F NMR** (376 MHz, CDCl_3_) δ −63.9. Compound characterization is in accordance with previous reports [53].

*o-Tolylmethanamine (**3i**)***;** The compound was prepared as described in the general procedure (colorless oil, yield = 118 mg, 99%); **^1^H NMR** (400 MHz, CDCl_3_) δ 7.31 (d, *J* = 6.6 Hz, 1H), 7.24–7.14 (m, 3H), 3.86 (s, 2H), 2.34 (s, 3H), 1.69 (s, 1H). **^13^C NMR** (101 MHz, CDCl_3_) δ140.9, 135.4, 130.2, 127.0, 126.7, 126.1, 43.9, 18.7. Compound characterization is in accordance with previous reports [53].

*4-Methylbenzyl amine (**3j**)***;** The compound was prepared as described in the general procedure (colorless oil, yield = 104 mg, 86%); **^1^H NMR** (400 MHz, CDCl_3_) δ 7.19 (d, *J* = 8.0 Hz, 2H), 7.15 (d, *J* = 5.4 Hz, 2H), 3.81 (s, 2H), 2.33 (s, 3H), 1.54–1.44 (m, 2H). **^13^C NMR** (101 MHz, CDCl_3_) δ140.3, 136.2, 129.1, 126.9, 46.2, 21.0. Compound characterization is in accordance with previous reports [51].

*4-Methoxybenzylamine (**3k**)***;** The compound was prepared as described in the general procedure (Colorless oil, yield = 118 mg, 86%); **^1^H NMR** (400 MHz, CDCl_3_) δ 7.22 (d, *J* = 8.6 Hz, 2H), 6.87 (d, *J* = 8.6 Hz, 2H), 3.79 (s, 5H), 1.43 (s, 2H). **^13^C NMR** (101 MHz, CDCl_3_) δ158.4, 135.5, 128.2, 113.8, 55.2, 45.8. Compound characterization is in accordance with previous reports.

*4-(Aminomethyl)-N,N-dimethylaniline (**3l**)***;** The compound was prepared as described in the general procedure (yellow oil, yield = 77mg, 51%); **^1^H NMR** (400 MHz, CDCl_3_) δ 7.18 (d, *J* = 8.6 Hz, 2H), 6.72 (d, *J* = 8.7 Hz, 2H), 3.76 (s, 2H), 2.93 (s, 6H), 1.47 (s, 2H). **^13^C NMR** (101 MHz, CDCl_3_) δ149.7, 131.5, 127.9, 112.8, 45.9, 40.7. Compound characterization is in accordance with previous reports [54].

*Naphthalen-1-ylmethanamine (**3m**)***;** The compound was prepared as described in the general procedure (yellow oil, yield = 130 mg, 83%); **^1^H NMR** (400 MHz, CDCl_3_) δ 8.07 (s, 1H), 7.89 (d, *J* = 7.8 Hz, 1H), 7.78 (d, *J* = 8.1 Hz, 1H), 7.59–7.43 (m, 4H), 4.32 (s, 2H), 1.59 (s, 2H). **^13^C NMR** (101 MHz, CDCl_3_) δ138.9, 133.8, 131.1, 128.8, 127.5, 126.1, 125.6, 125.5, 124.4, 123.1, 44.0. Compound characterization is in accordance with previous reports [53].

*Thiophen-2-ylmethanamine (**3n**)***;** The compound was prepared as described in the general procedure (yellow oil, yield = 82 mg, 73%); **^1^H NMR** (400 MHz, CDCl_3_) δ 7.18 (d, *J* = 5.0 Hz, 1H), 6.94 (t, *J* = 4.2 Hz, 1H), 6.90 (d, *J* = 2.9 Hz, 1H), 4.03 (s, 2H), 1.65 (s, 2H). **^13^C NMR** (101 MHz, CDCl_3_) δ 147.4, 126.7, 123.9, 123.5, 41.3.

*Octan-1-amine (**3o**)***;** The compound was prepared as described in the general procedure (colorless oil, yield = 115 mg, 89%); **^1^H NMR** (400 MHz, CDCl_3_) δ 2.65 (t, *J* = 8.0 Hz, 2H), 1.45–1.20 (m, 14H), 0.87 (t, *J* = 8.0 Hz, 3H). **^13^C NMR** (101 MHz, CDCl_3_) δ42.1, 33.8, 31.7, 29.4, 29.2, 26.8, 22.5, 14.0. Compound characterization is in accordance with previous reports [55].

*Dodecan-1-amine (**3p**)***;** The compound was prepared as described in the general procedure (colorless oil, yield = 170 mg, 92%); **^1^H NMR** (400 MHz, CDCl_3_) δ 2.66 (t, *J* = 6.9 Hz, 2H), 1.41 (s, 2H), 1.24 (s, 20H), 0.86 (t, *J* = 6.4 Hz, 3H). **^13^C NMR** (101 MHz, CDCl_3_) δ42.2, 33.8, 31.8, 29.54, 29.53, 29.41, 29.41, 29.24, 29.24, 26.8, 22.6, 14.0. Compound characterization is in accordance with previous reports [52].

*Cyclohexylmethanamine (**3q**)***;** The compound was prepared as described in the general procedure (colorless oil, yield = 71 mg, 63%); **^1^H NMR** (400 MHz, CDCl_3_) δ 2.49 (d, *J* = 6.3 Hz, 2H), 1.75–1.62 (m, 6H), 1.29–1.18 (m, 5H), 0.87 (t, *J* = 11.8 Hz, 2H). **^13^C NMR** (101 MHz, CDCl_3_) δ48.8, 41.2, 30.7, 26.6, 25.9. Compound characterization is in accordance with previous reports [52].

*Phenethylamine (**3r**)***;** The compound was prepared as described in the general procedure (yellow oil, yield = 114 mg, 94%); **^1^H NMR** (400 MHz, CDCl_3_) δ 7.34–7.25 (m, 2H), 7.23–7.18 (m, 3H), 2.97 (t, *J* = 7.1 Hz, 2H), 2.77 (t, *J* = 6.9 Hz, 2H), 2.13 (s, 2H). **^13^C NMR** (101 MHz, CDCl_3_) δ139.5, 128.7, 128.4, 126.1, 43.2, 39.5. Compound characterization is in accordance with previous reports [56].

*2-(4-Fluorophenyl)ethan-1-amine****(3s)*;** The compound was prepared as described in the general procedure (yellow oil, yield = 130 mg, 94%); **^1^H NMR** (400 MHz, CDCl_3_) δ 7.14 (d, *J* = 13.4 Hz, 2H), 6.97 (d, *J* = 17.4 Hz, 2H), 2.93 (s, 2H), 2.71 (s, 2H). **^13^C NMR** (101 MHz, CDCl_3_) δ160.2, 135.4, 130.0, 115.2, 115.0, 43.5, 39.1. **^19^F NMR** (376 MHz, CDCl_3_) δ −118.8. Compound characterization is in accordance with previous reports [56].

*2-(4-Methoxyphenyl)ethanamine (**3t**)***;** The compound was prepared as described in the general procedure (yellow oil, yield = 148 mg, 98%); **^1^H NMR** (400 MHz, CDCl_3_) δ 7.10 (d, *J* = 8.3 Hz, 2H), 6.83 (d, *J* = 8.2 Hz, 2H), 3.77 (s, 3H), 2.91 (s, 2H), 2.68 (s, 2H), 1.32 (s, 2H). **^13^C NMR** (101 MHz, CDCl_3_) δ157.9, 131.7, 129.6, 114.4, 113.8, 55.1, 43.6, 39.0. Compound characterization is in accordance with previous reports [51].

*2-(2,4-difluorophenyl)ethan-1-amine (**3u**)***;** The compound was prepared as described in the general procedure (yellow oil, yield = 141 mg, 90%); **^1^H NMR** (400 MHz, CDCl_3_) δ 7.18–7.09 (m, 1H), 6.82–6.73 (m, 2H), 2.91 (t, *J* = 6.9 Hz, 2H), 2.72 (t, *J* = 6.9 Hz, 2H), 1.24 (s, 2H). **^13^C NMR** (101 MHz, CDCl_3_) δ160.3, 159.9, 131.5, 131.4, 131.3, 122.5, 122.3, 111.0, 110.8, 103.9, 103.6, 103.3, 42.3, 32.8. **^19^F NMR** (376 MHz, CDCl_3_) δ −114.8, −115.9.

*2,2-Diphenylethan-1-amine (***3v***)***;** The compound was prepared as described in the general procedure (colorless, yield = 187 mg, 95%); **^1^H NMR** (400 MHz, CDCl_3_) δ 7.33–7.20 (m, 10H), 3.99 (t, *J* = 7.6 Hz, 1H), 3.33 (d, *J* = 7.6 Hz, 2H), 1.22 (s, 2H). **^13^C NMR** (101 MHz, CDCl_3_) δ142.7, 128.5, 128.0, 126.4, 55.1, 47.0.

## 4. Conclusions

In conclusion, we have developed a simple protocol for the reduction of nitriles to afford primary amines using ammonia-borane as the reductant in the presence of one molar equivalent of TiCl_4_ in diethyl ether at room temperature. A broad range of aromatic, heteroaromatic, benzylic, and aliphatic nitriles were efficiently reduced under this condition in moderate to very high yields. This reducing system affords negligible side products, and the workup of the reaction mixture is very simple. The reaction is believed to progress via the activation of the nitrile by titanium tetrachloride, followed by the hydroboration of the carbon nitrogen triple bond.

## Figures and Tables

**Table 1:** Optimization of reaction conditions for the reduction of benzonitrile.

Entry	LA	R_3_N-BH_3_	LA: R_3_N-BH_3_	Solvent	Time, h	Product Yield *^a^*, %
1	none	**1a**	0:2	Et_2_O	24	NR *^b^*
2	none	**1a**	0:1	THF	20	24 *^c^*
3	none	**1a**	0:2	THF	20	60 *^c^*
4	TiCl_4_	**1a**	0.7:2	Et_2_O	3	77
**5 *^d^***	**TiCl_4_**	**1a**	**1:2**	**Et_2_O**	**1**	**95**
6	TiCl_4_	**1a**	1:1.5	Et_2_O	24	71
7	TiCl_4_	**1a**	0.7:2	CH_2_Cl_2_	3	71
8	TiCl_4_	**1a**	0.7:2	THF	3	20
9	TiCl_4_	**1a**	0.7:2	pentane	3	NR *^b^*
10	TiBr_4_	**1a**	0.5:2	Et_2_O	3	95
11	HfCl_4_	**1a**	0.7:2	Et_2_O	3	63
12	BF_3_.OEt_2_	**1a**	0.7:2	Et_2_O	3	17
13	AlCl_3_	**1a**	0.7:2	Et_2_O	3	65
14	FeCl_3_	**1a**	0.7:2	Et_2_O	3	55
15	TiCl_4_	**1b**	1:2	Et_2_O	3	16
16	TiCl_4_	**1c**	1:2	Et_2_O	3	17
17	TiCl_4_	**1d**	1:2	Et_2_O	3	NR *^b^*
18	TiCl_4_	**1e**	1:2	Et_2_O	3	34

*^a^* isolated yield. *^b^* NR = no reaction. *^c^*determined as a mixture of **3a** and **2a** by PMR after workup. *^d^* optimal condition.

## Data Availability

All of the ^1^H, ^11=9^F, and ^13^C NMR spectra are available in the supporting information.

## Supplementary Materials

The following supporting information can be downloaded at: https://www.mdpi.com/article/10.3390/molecules28010060/s1, NMR spectra of product amines.

## Sample Availability

Samples of the compounds are not available from the authors.
