# Supplementary material for: Room Temperature Reduction of Titanium Tetrachloride-Activated Nitriles to Primary Amines with Ammonia-Borane
Source: Molecules. 2022 Dec 21;28(1):60. doi: 10.3390/molecules28010060 (PMC9822325; doi:10.3390/molecules28010060)
Supplement: Supplementary file 1 [file molecules-28-00060-s001.zip › molecules-2116500-supplementary.pdf]

SUPPORTING INFORMATION:

Room temperature reduction of titanium tetrachloride-activated nitriles to primary amines with ammonia-borane

P. Veeraraghavan Ramachandran,* Abdulkhaliq A. Alawaed

† Herbert C. Brown Center for Borane Research, Department of Chemistry, Purdue University, West Lafayette, Indiana 47907, United States

E-mail: chandran@purdue.edu

Contents:

Page

NMR spectra of product amines S2-S29

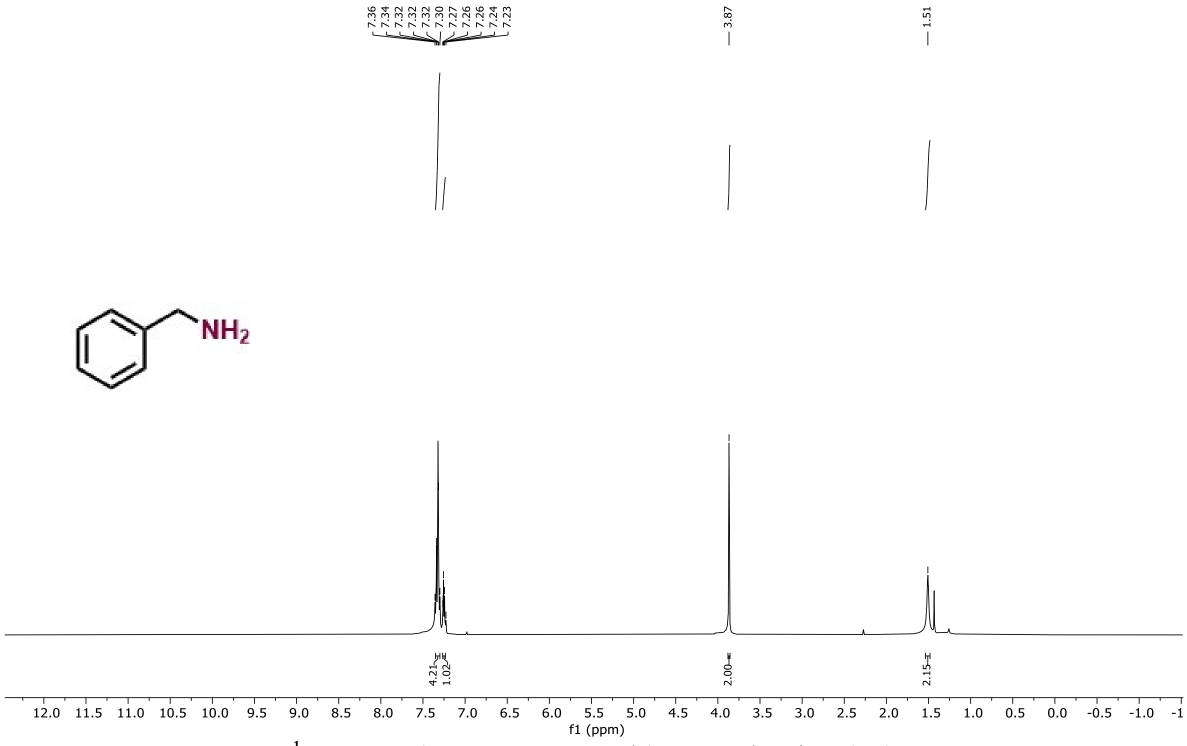

^{1}H NMR (400 MHz, $CDCl_3$) Benzylamine (**3a**)

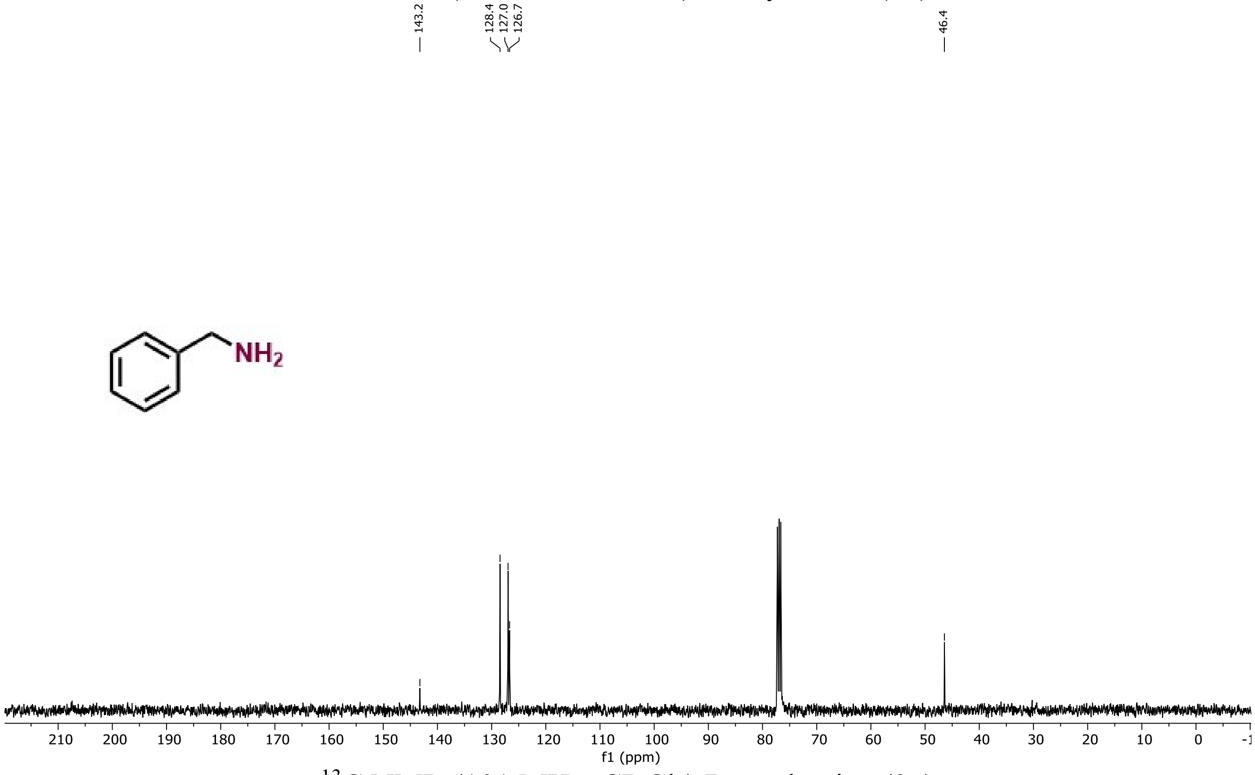

^{13}C NMR (101 MHz, $CDCl_3$) Benzylamine (**3a**)

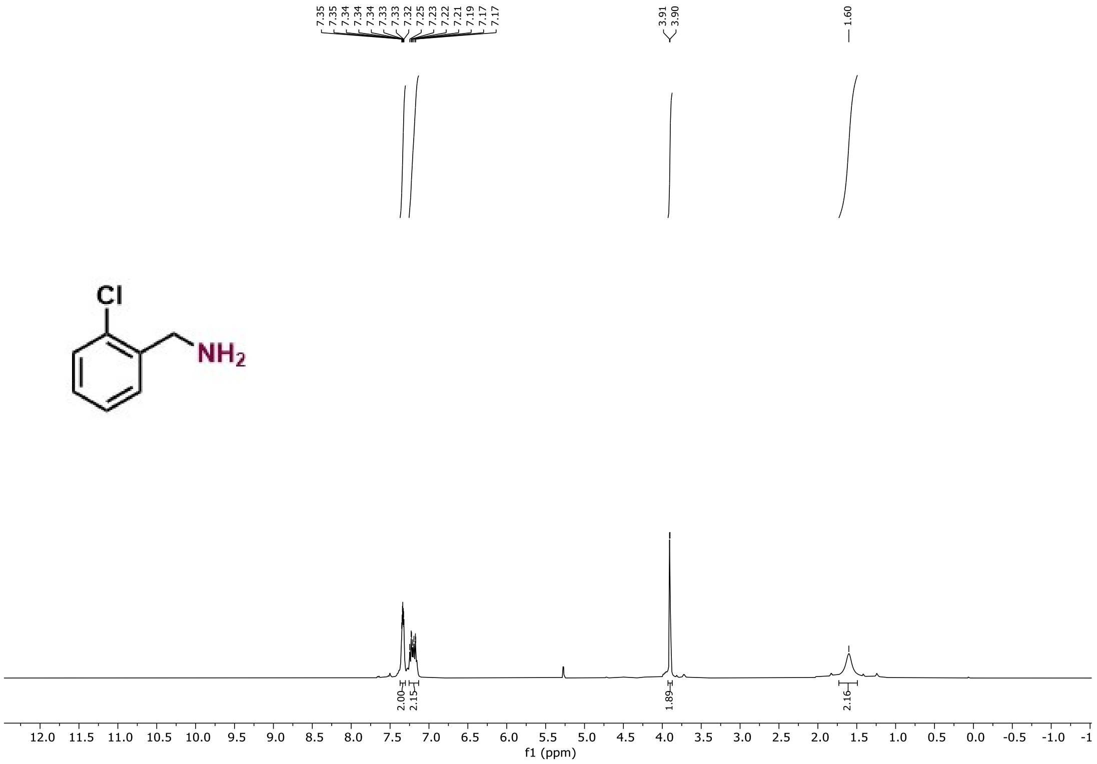

^{1}H NMR (400 MHz, $CDCl_3$) 2-Chlorobenzylamine (**3b**)

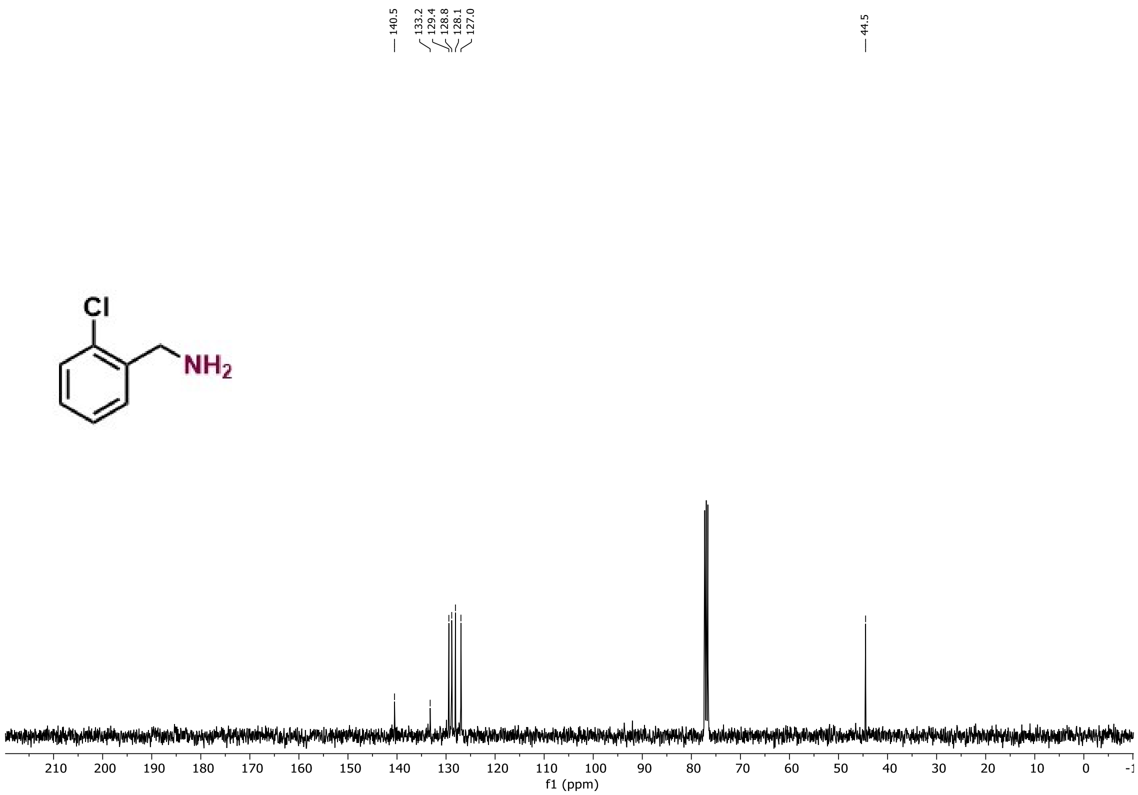

^{13}C NMR (101 MHz, $CDCl_3$) 2-Chlorobenzylamine (**3b**)

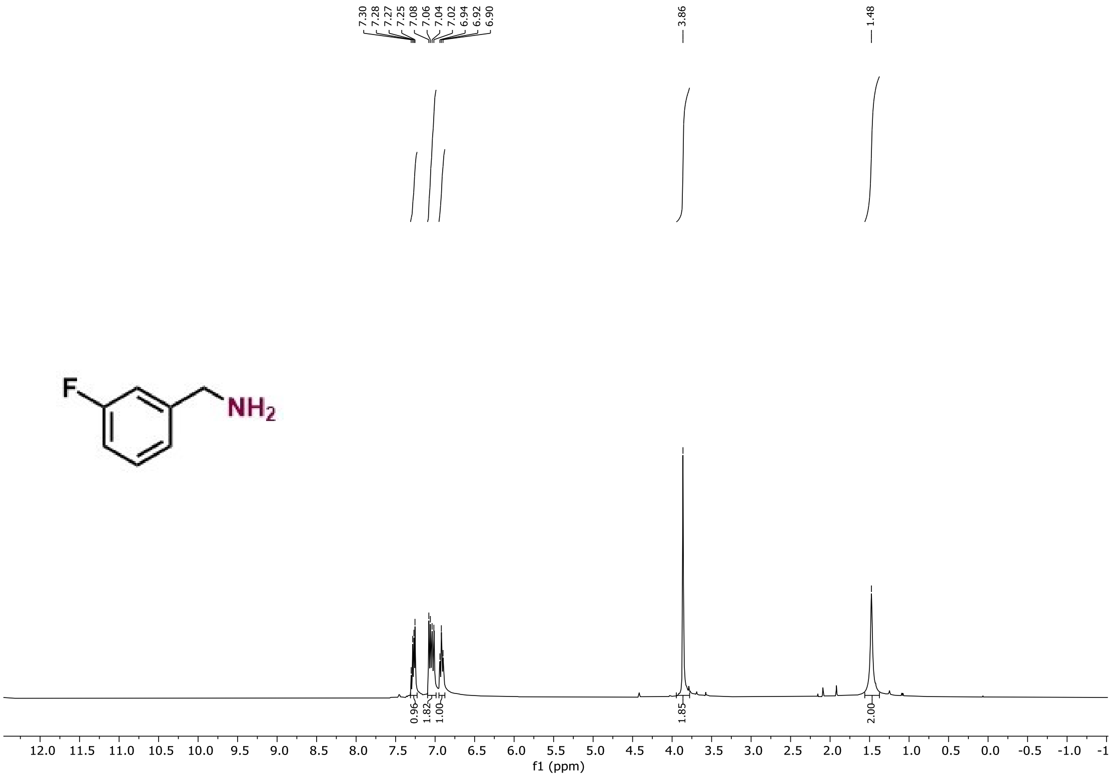

^{1}H NMR (400 MHz, $CDCl_3$) 3-Flourobenzylamine (**3c**)

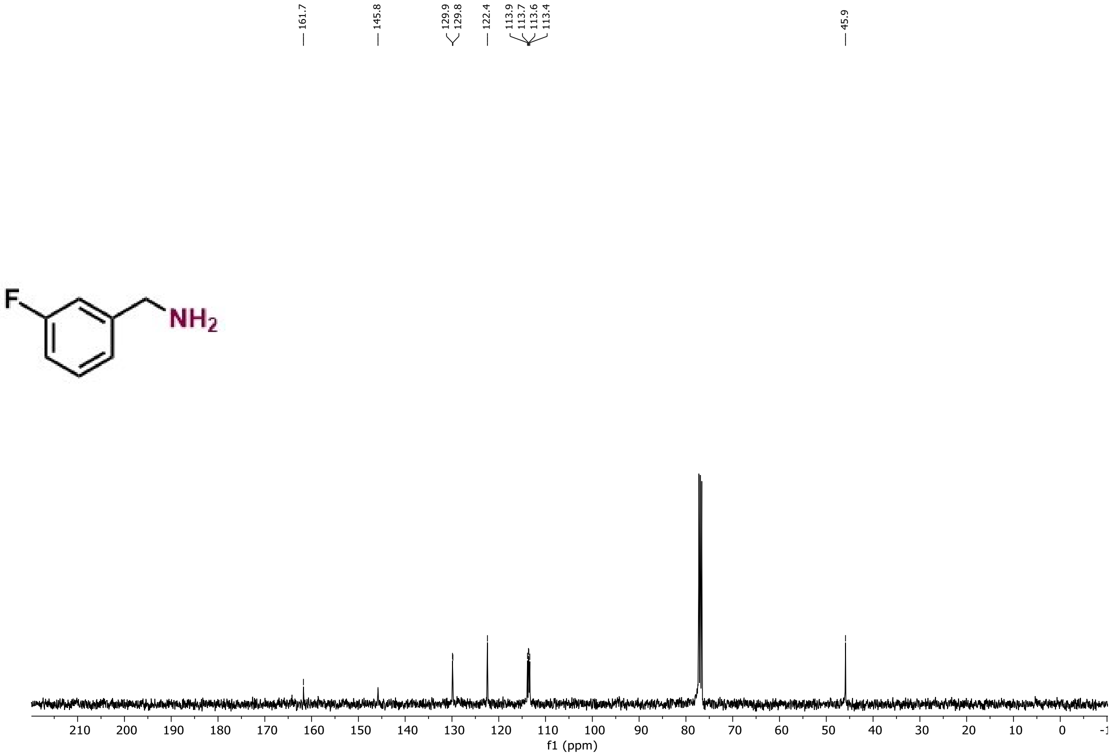

^{13}C NMR (101 MHz, $CDCl_3$) 3-Flourobenzylamine (**3c**)

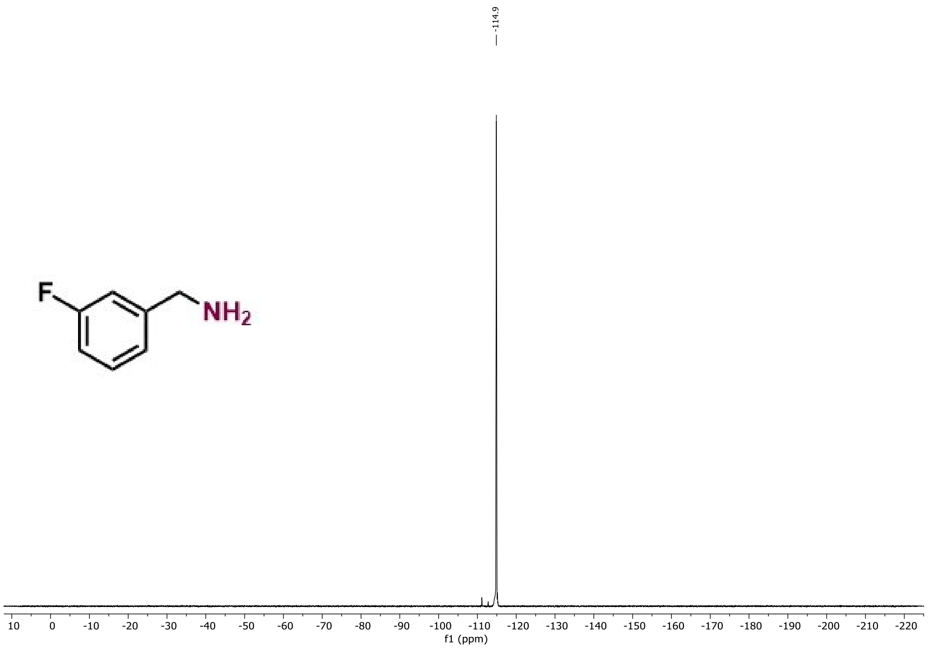

^{19}F NMR (282 MHz, $CDCl_3$) 3-Fluorobenzylamine (**3c**)

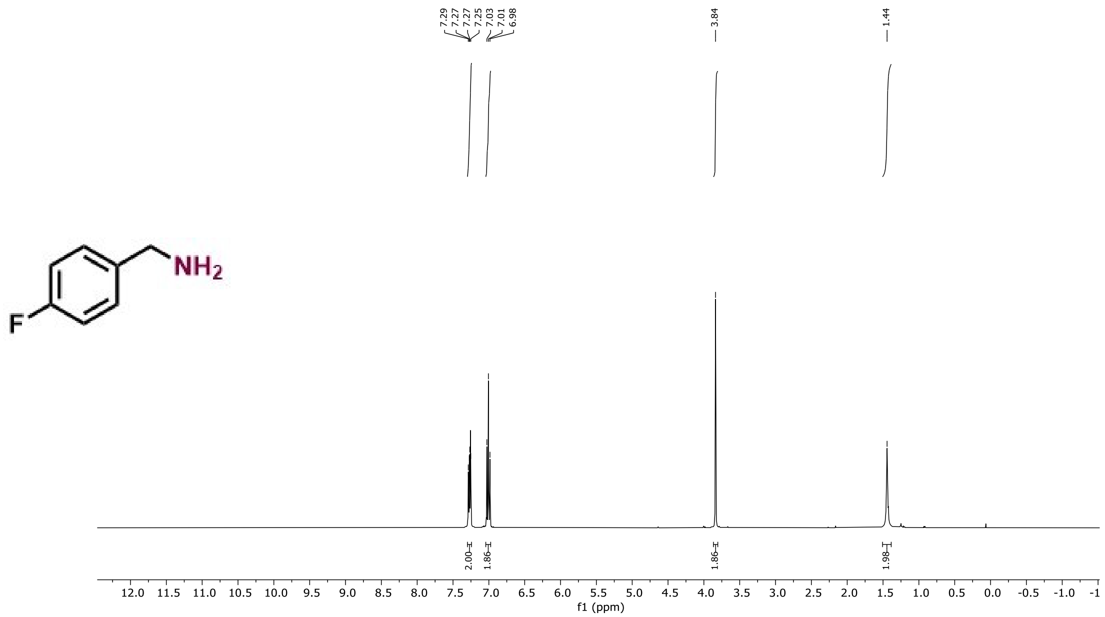

^{1}H NMR (400 MHz, $CDCl_3$) 4-Fluorobenzylamine (**3d**)

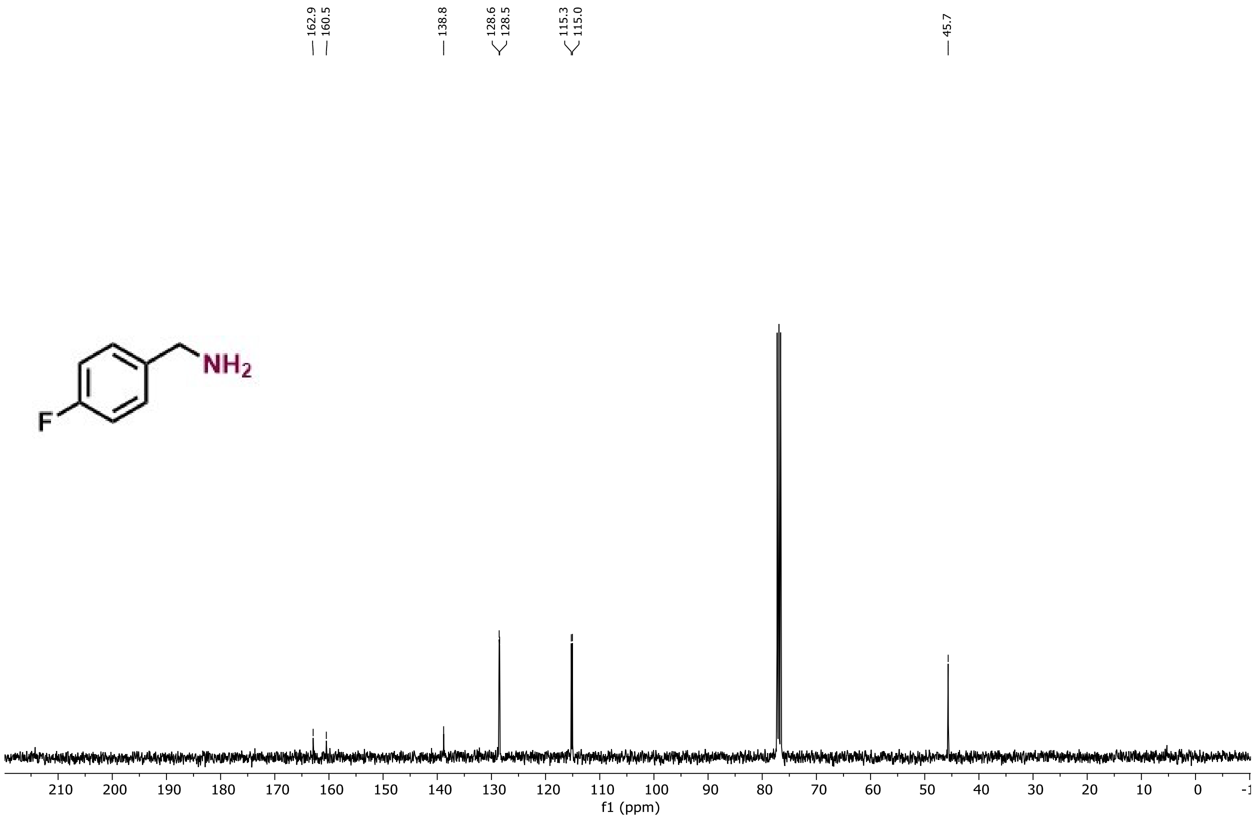

^{13}C NMR (101 MHz, $CDCl_3$) 4-Fluorobenzylamine (**3d**)

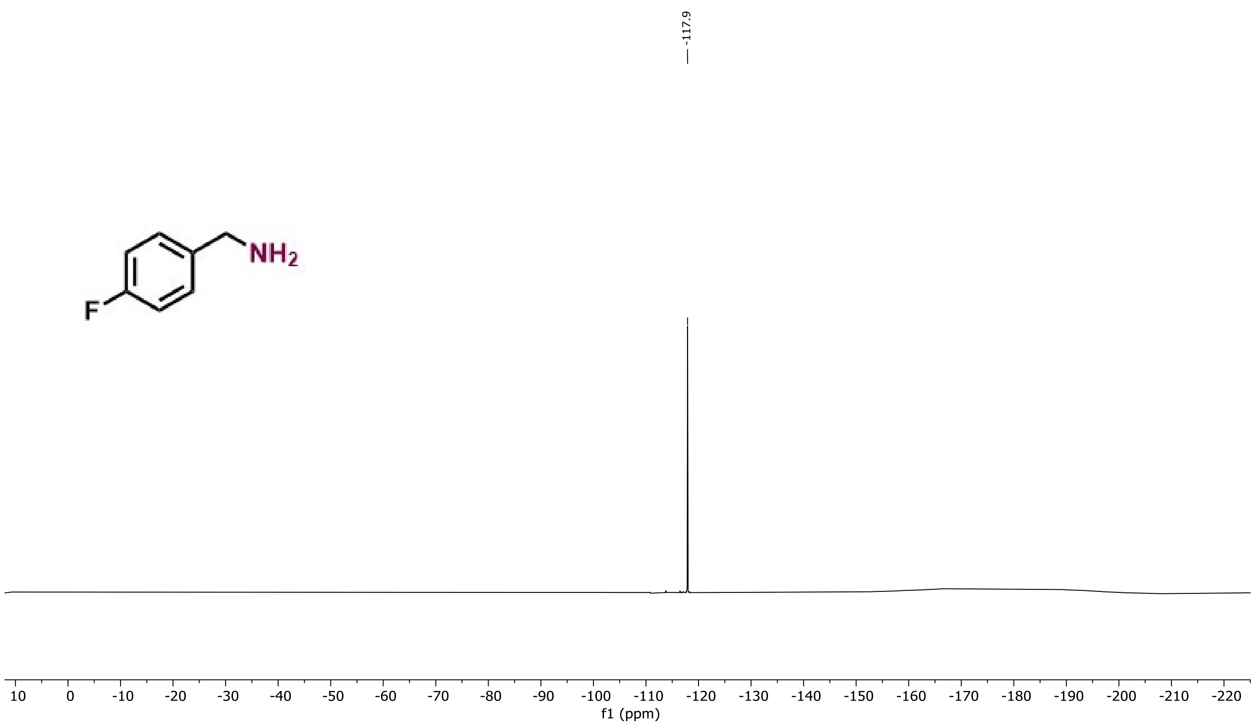

^{19}F NMR (282 MHz, CDCl$_3$) 4-Fluorobenzylamine (**3d**)

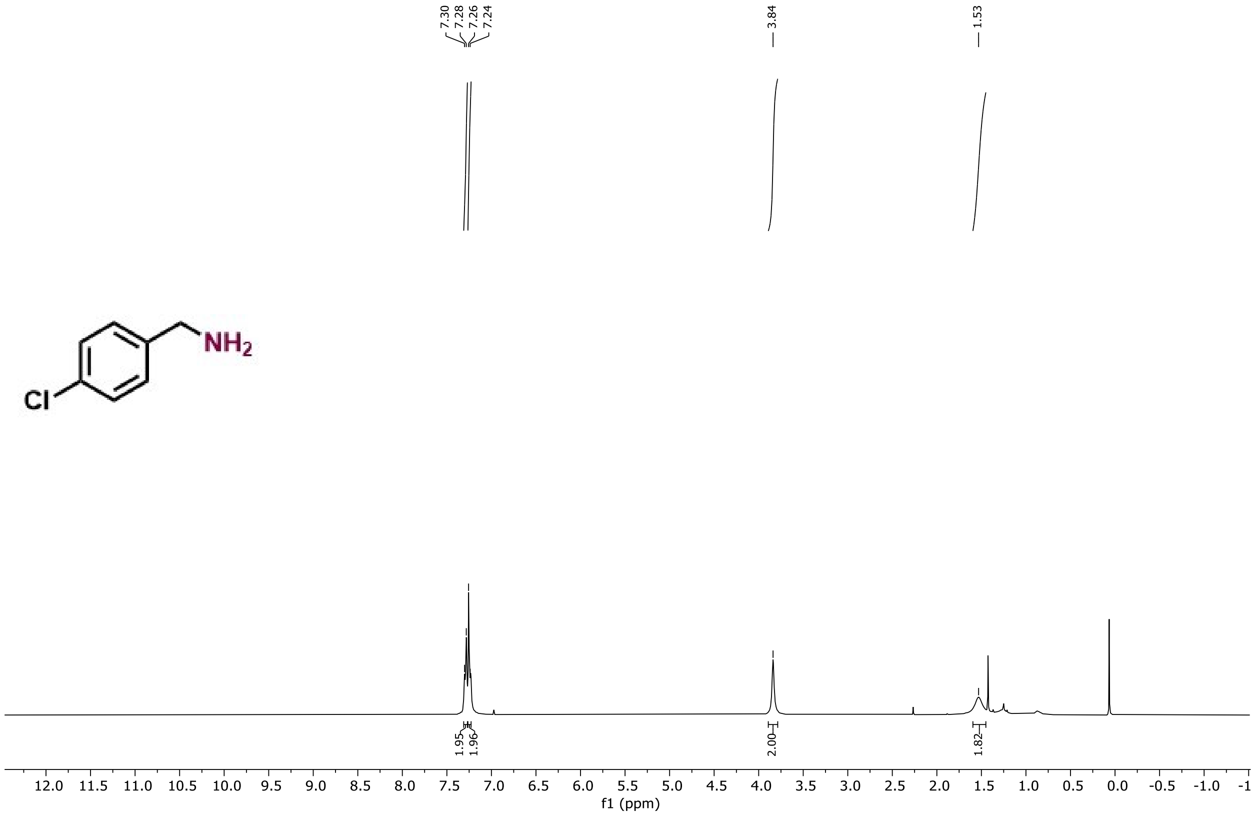

^{1}H NMR (400 MHz, CDCl$_3$) 4-Chlorobenzylamine (**3e**)

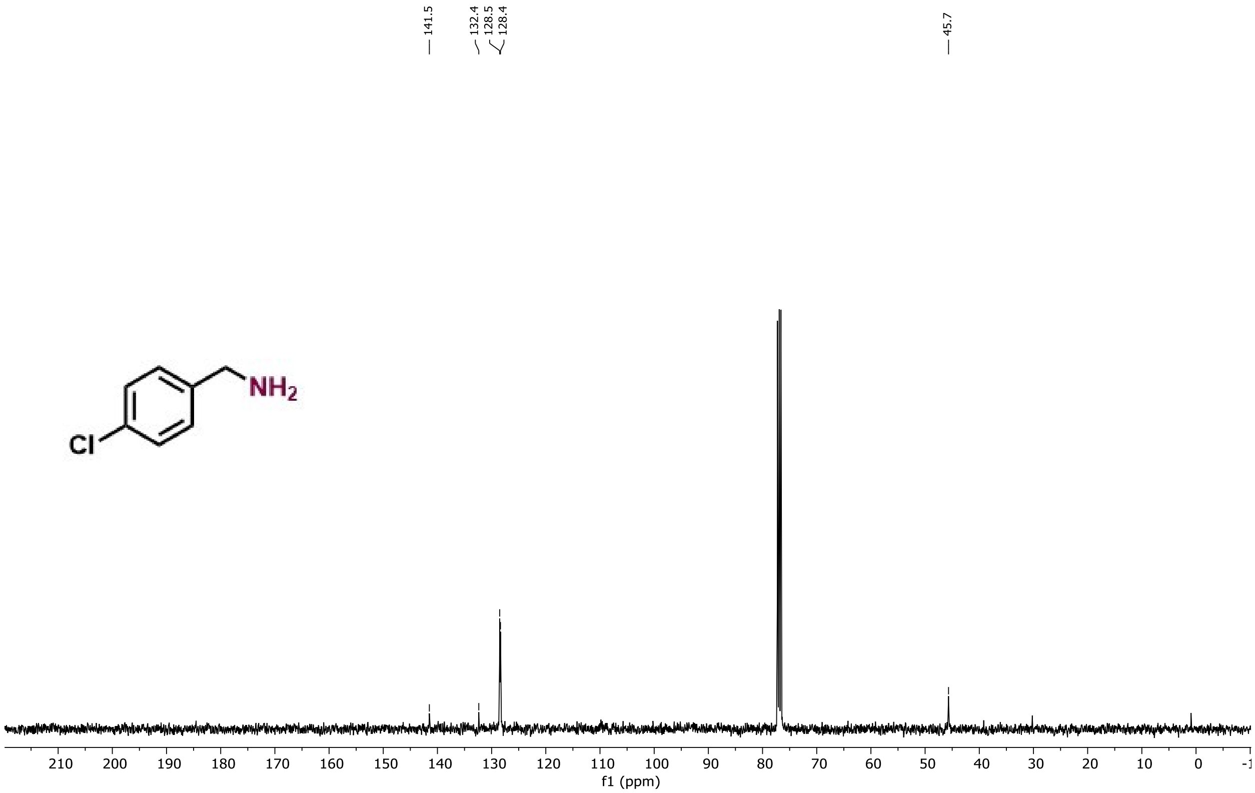

^{13}C NMR (101 MHz, $CDCl_3$) 4-Chlorobenzylamine (**3e**)

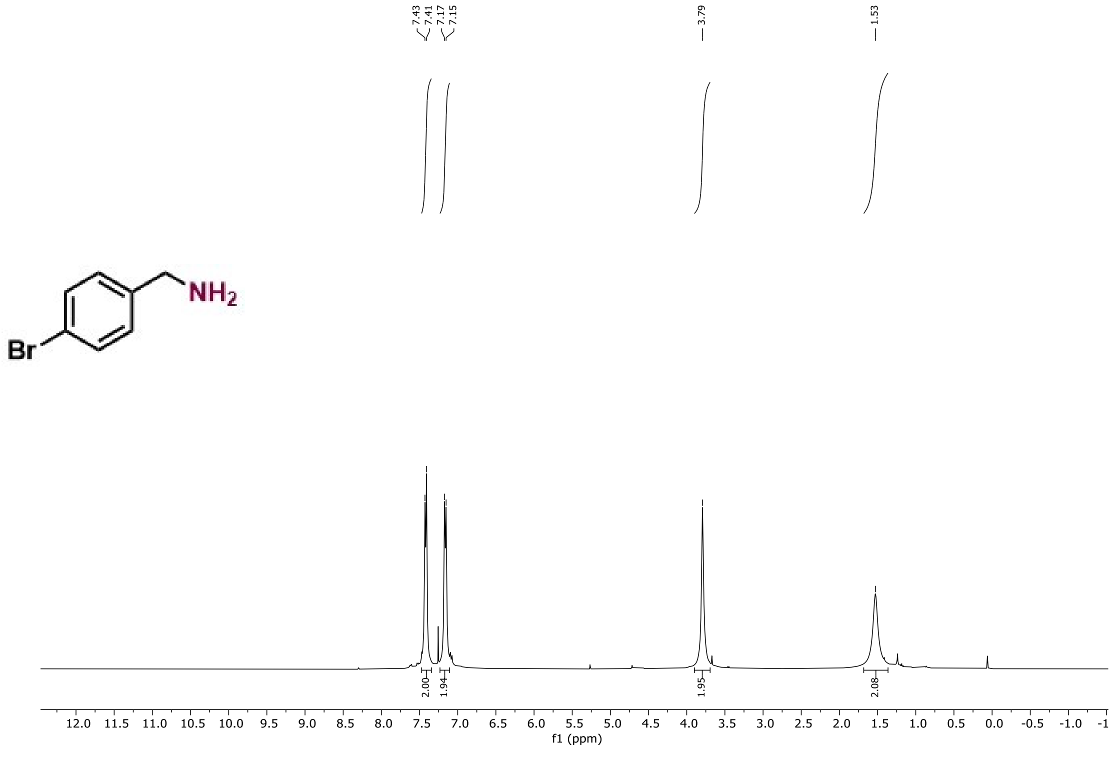

^{1}H NMR (400 MHz, $CDCl_3$) 4-Bromobenzylamine (**3f**)

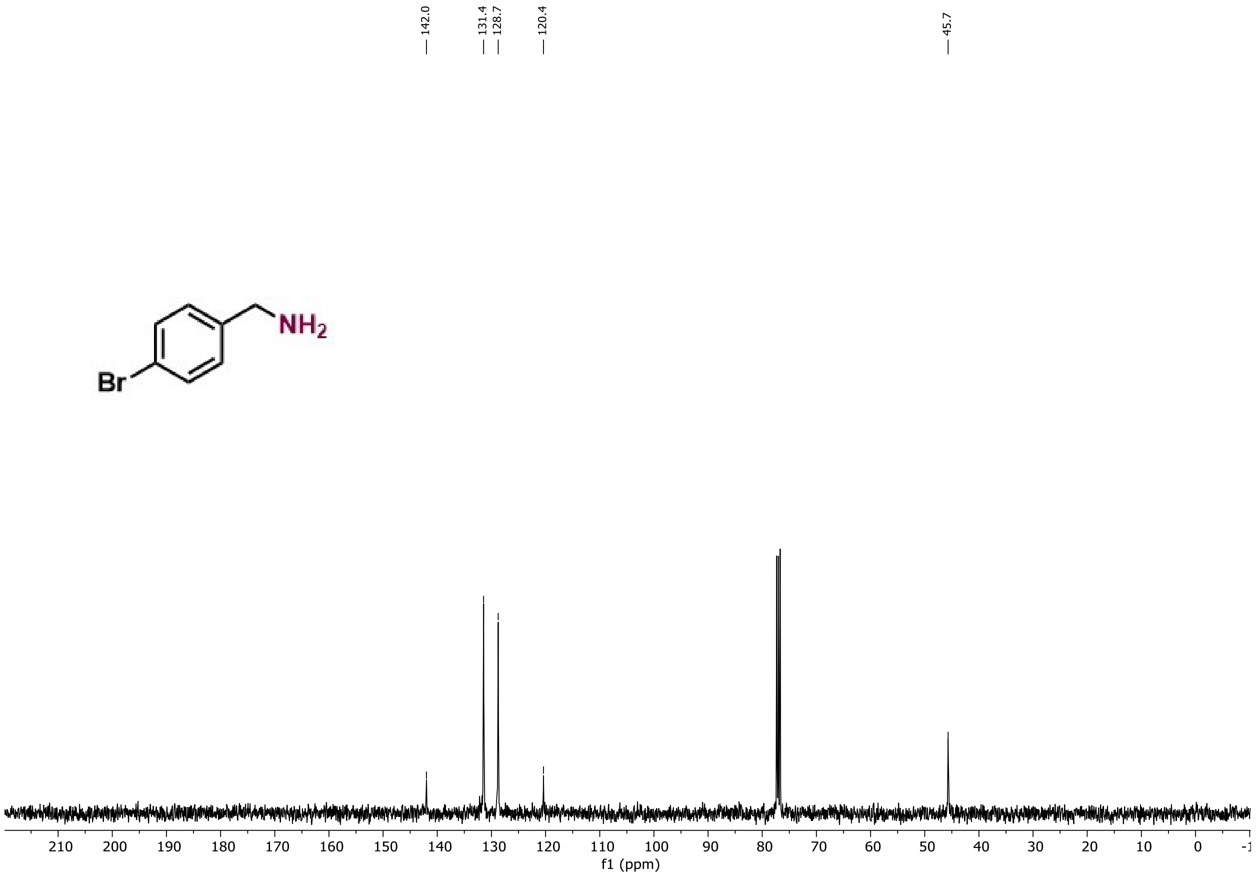

^{13}C NMR (101 MHz, $CDCl_3$) 4-Bromobenzylamine (**3f**)

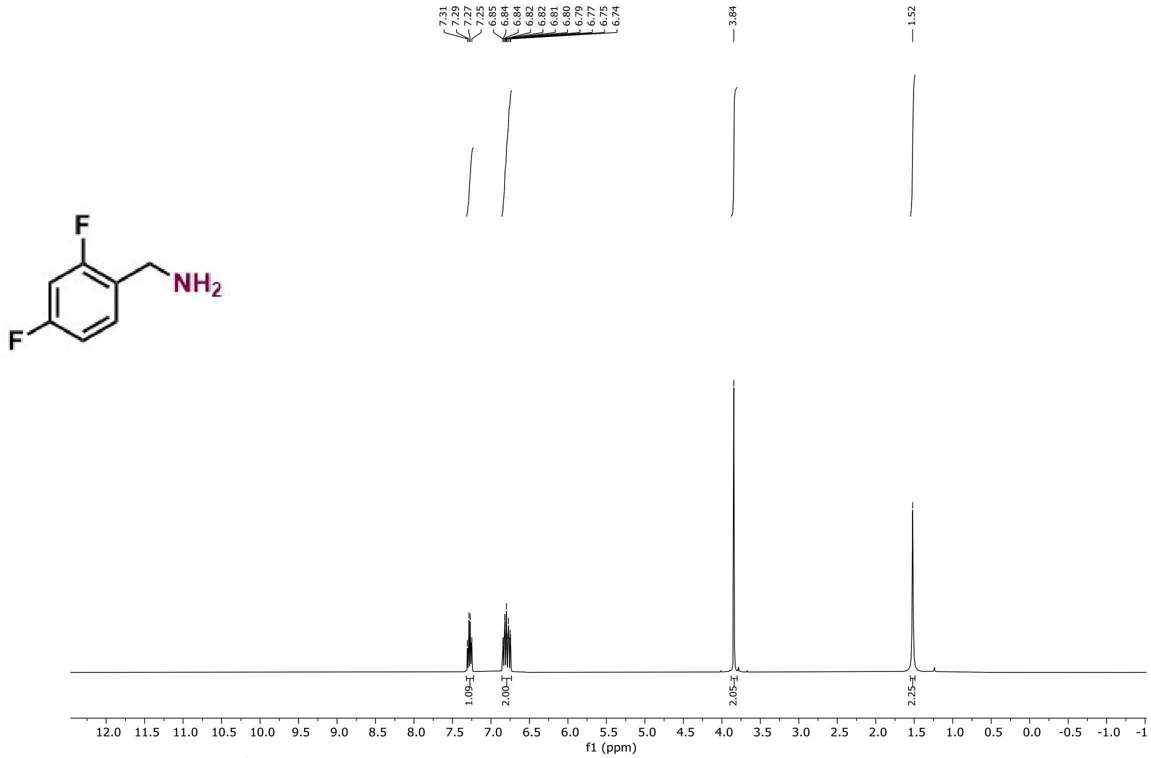

^{1}H NMR (400 MHz, $CDCl_3$) 2,4-Flourobenzylamine (**3g**)

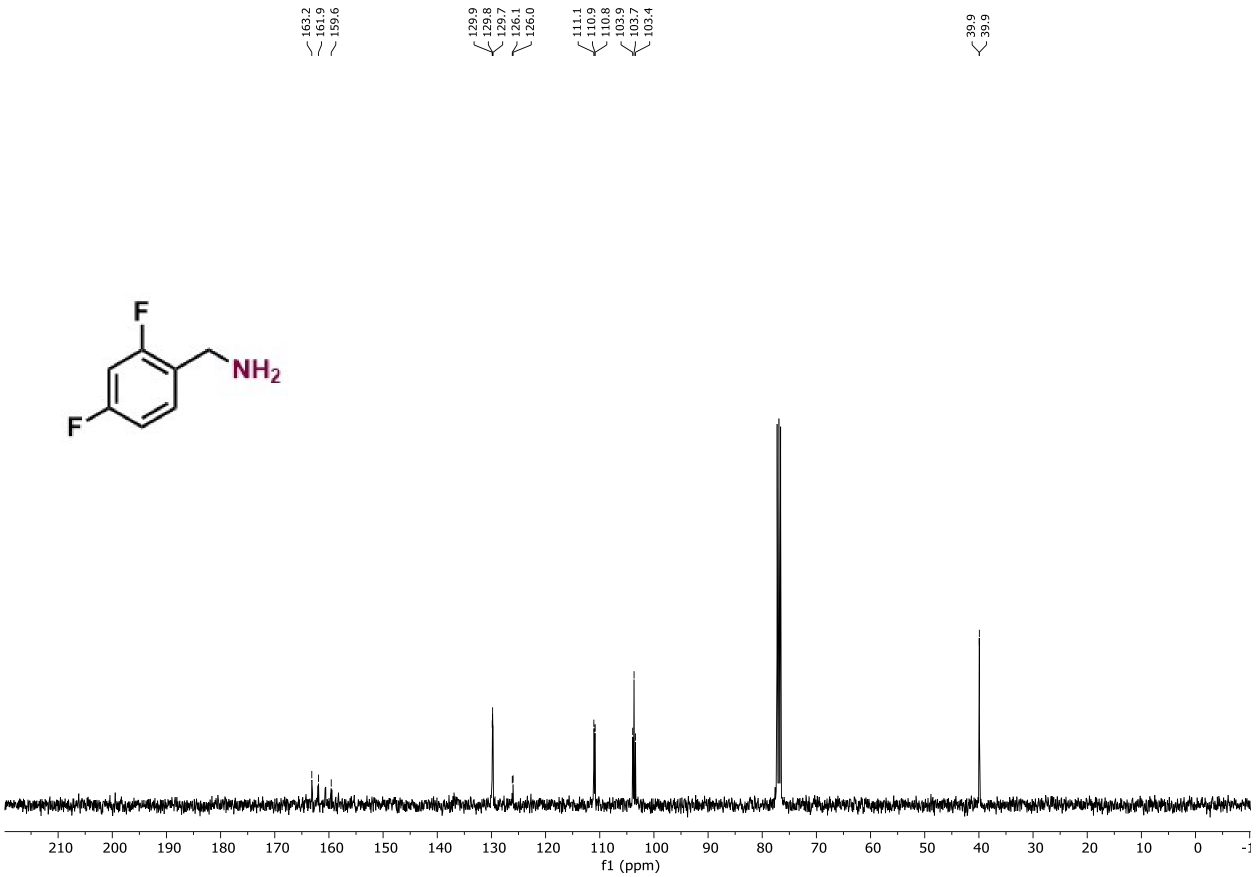

^{13}C NMR (101 MHz, $CDCl_3$) 2,4-Flourobenzylamine (**3g**)

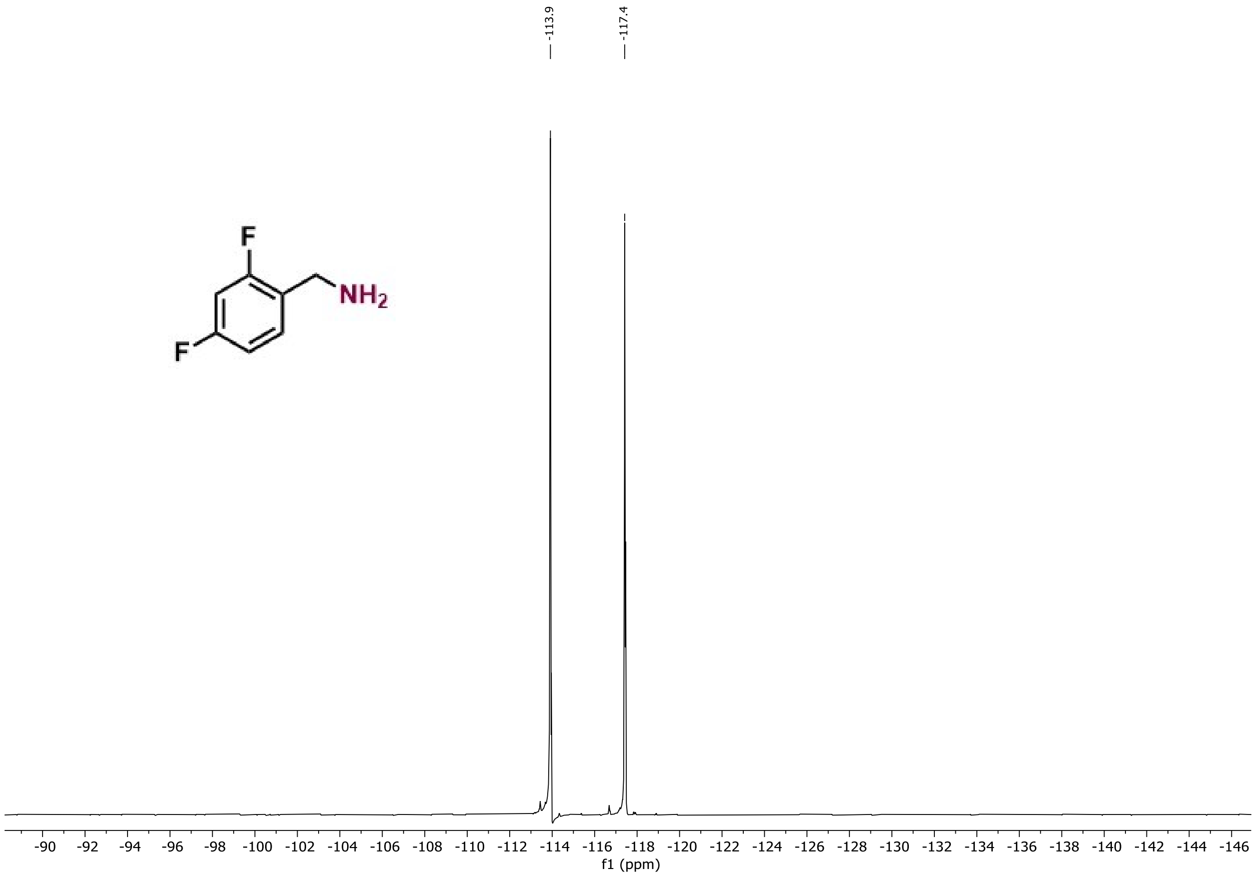

^{19}F NMR (282 MHz, $CDCl_3$) 2,4-Fluorobenzylamine (**3g**)

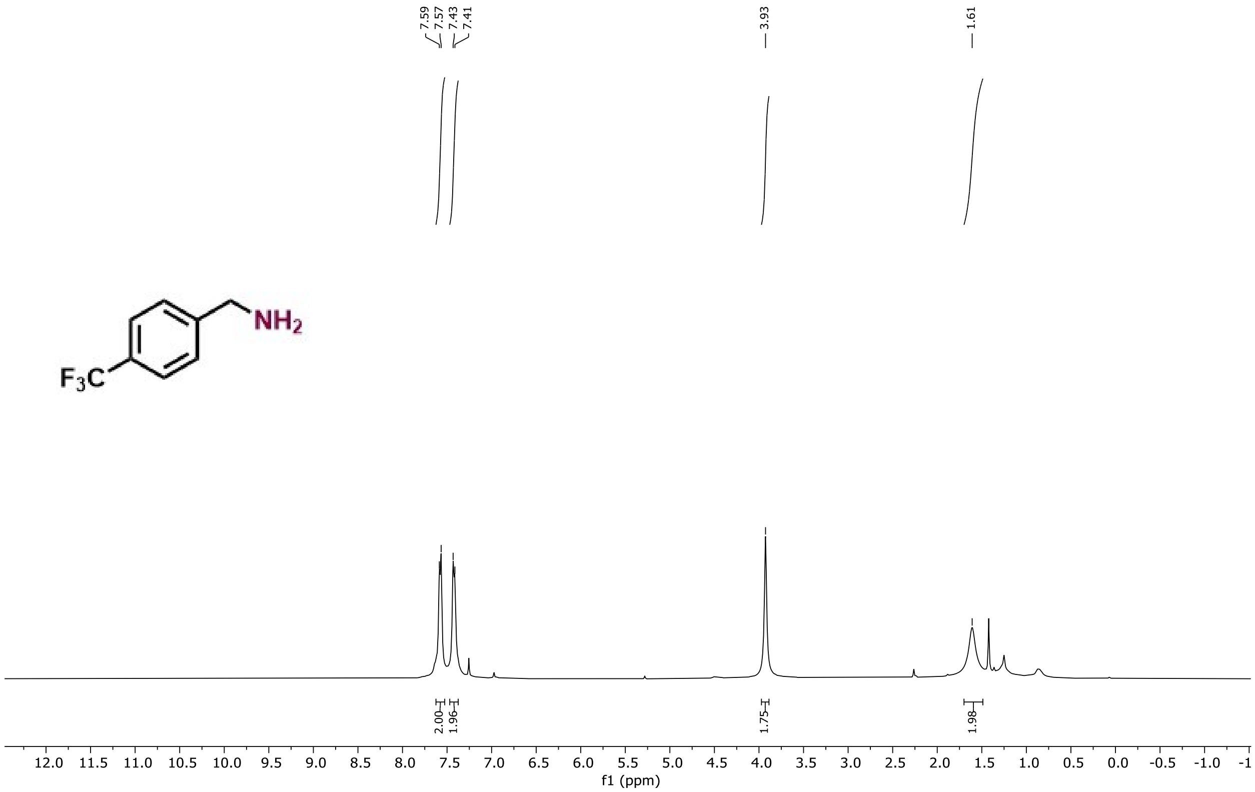

^{1}H NMR (400 MHz, $CDCl_3$) (4-(Trifluoromethyl)phenyl)methanamine (**3h**)

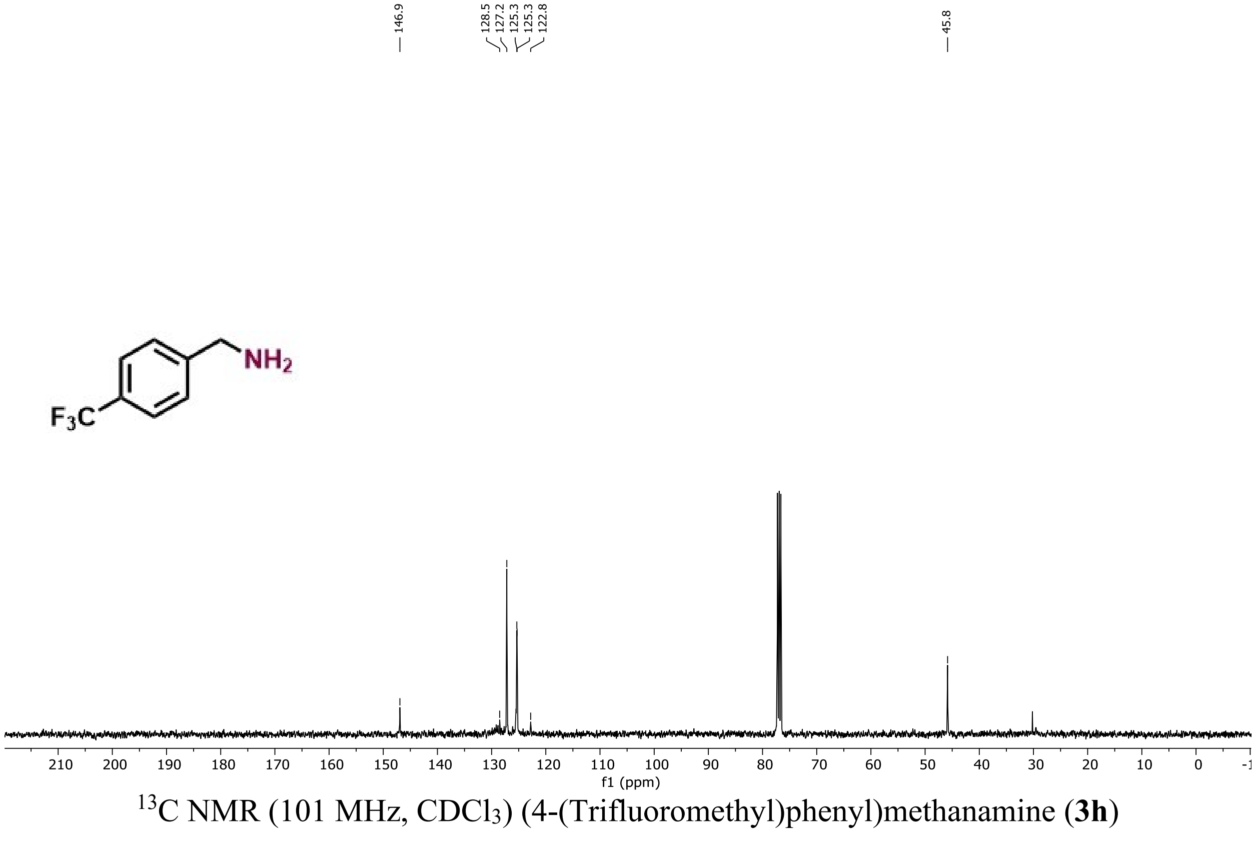

^{13}C NMR (101 MHz, $CDCl_3$) (4-(Trifluoromethyl)phenyl)methanamine (**3h**)

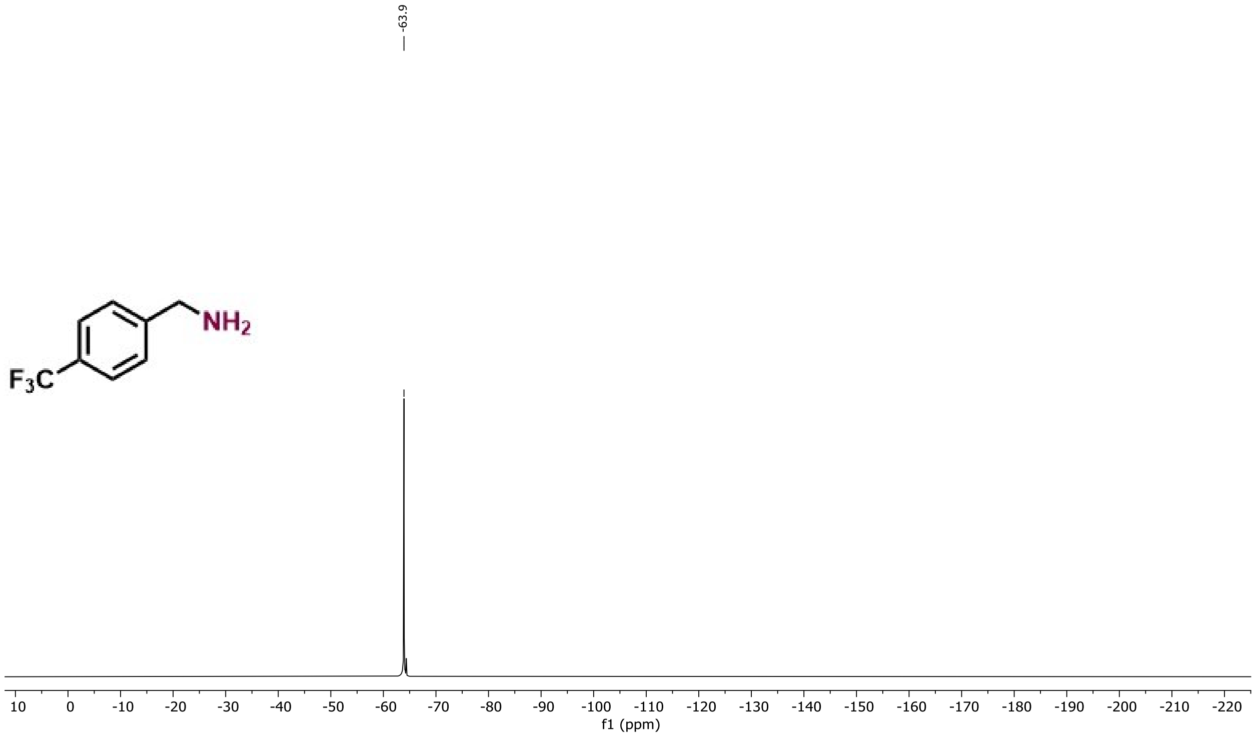

^{19}F NMR (282 MHz, $CDCl_3$) (4-(Trifluoromethyl)phenyl)methanamine (**3h**)

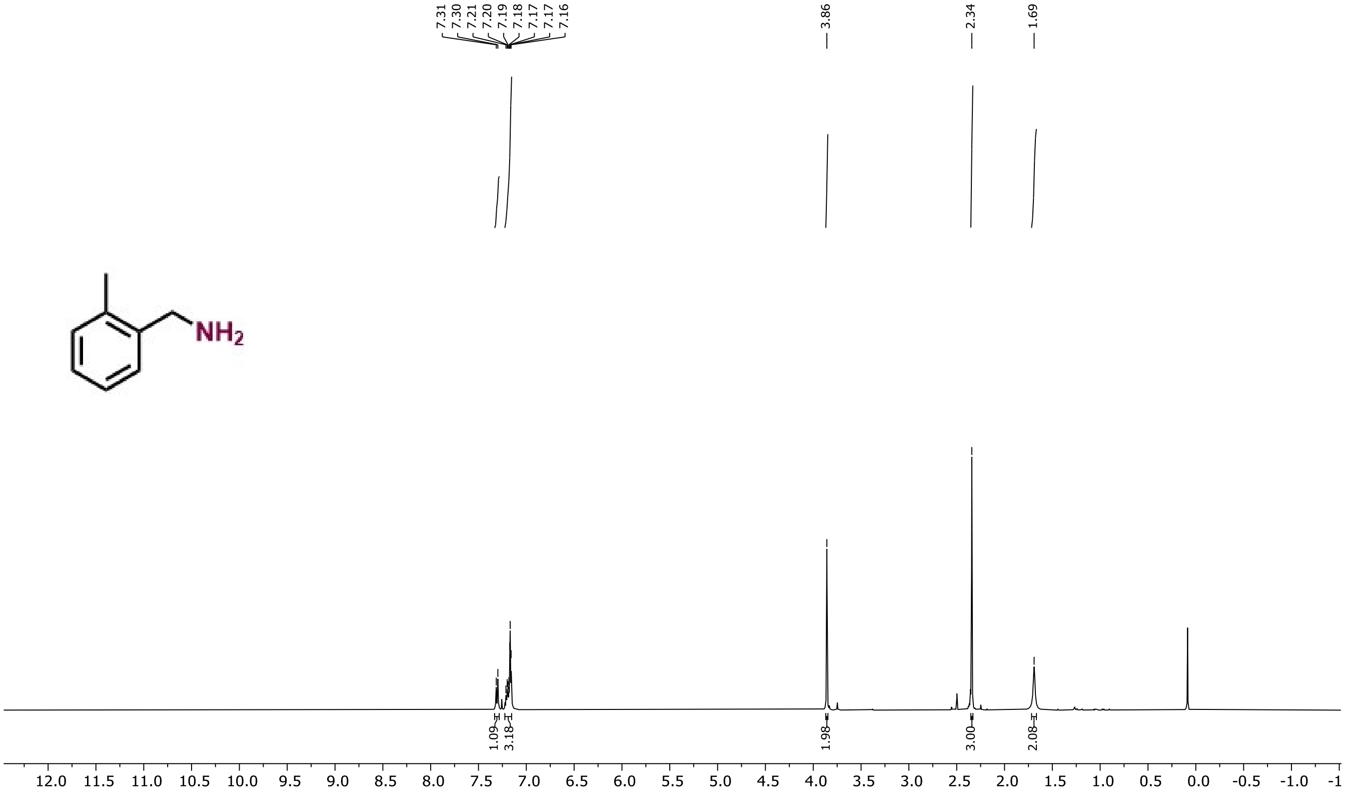

^{1}H NMR (400 MHz, $CDCl_3$) o-Tolylmethanamine (**3i**)

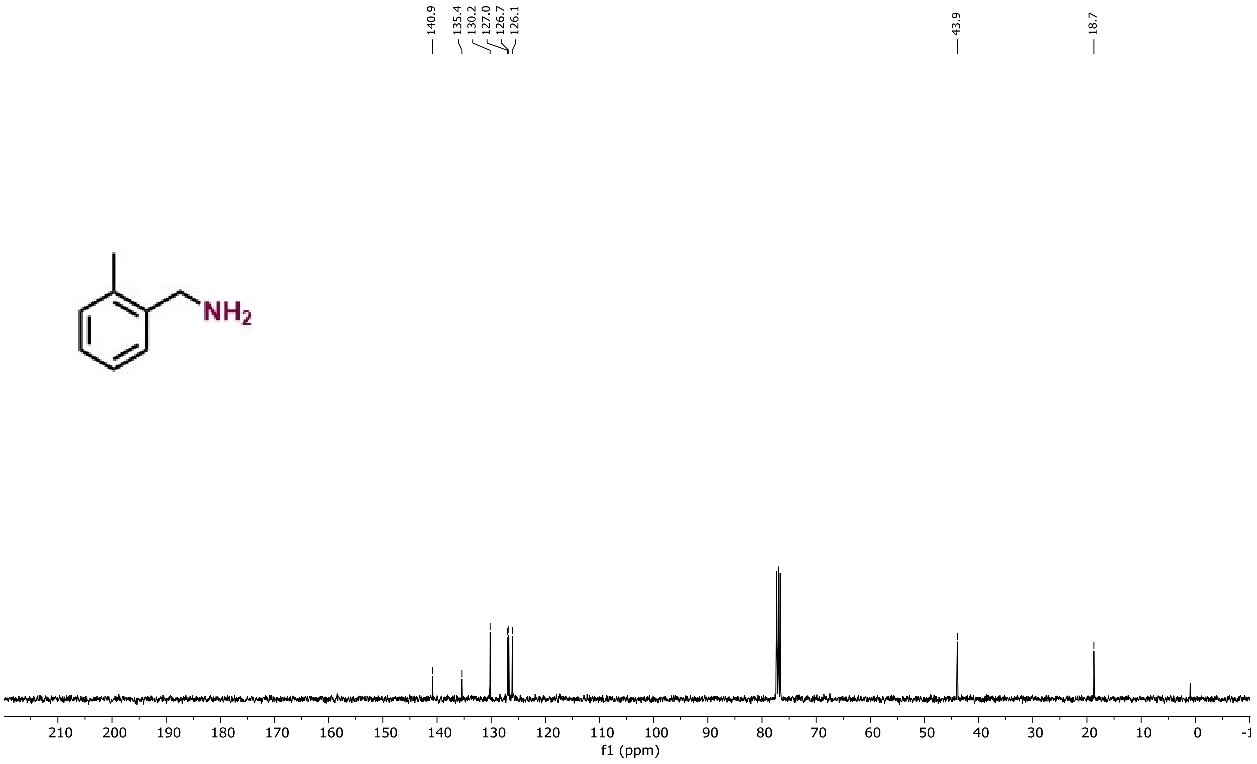

^{13}C NMR (101 MHz, $CDCl_3$) o-Tolylmethanamine (**3i**)

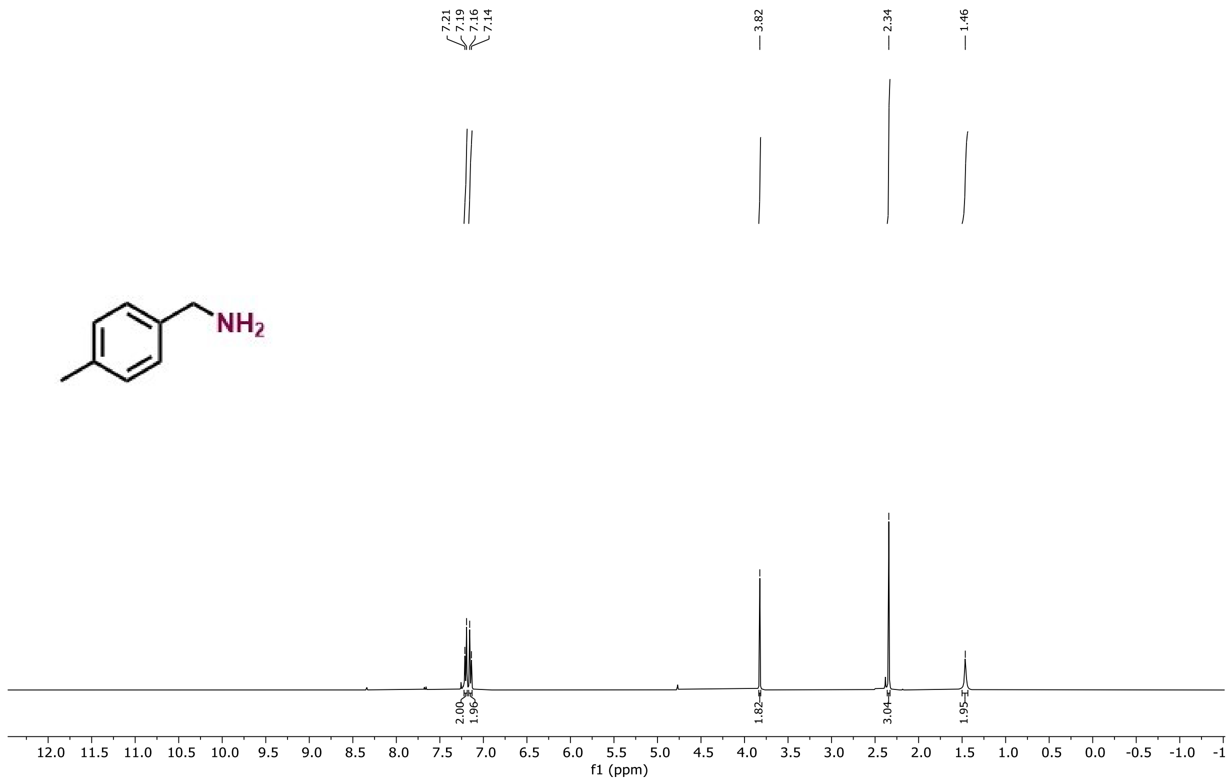

^{1}H NMR (400 MHz, $CDCl_3$) 4-Methylbenzyl amine (**3j**)

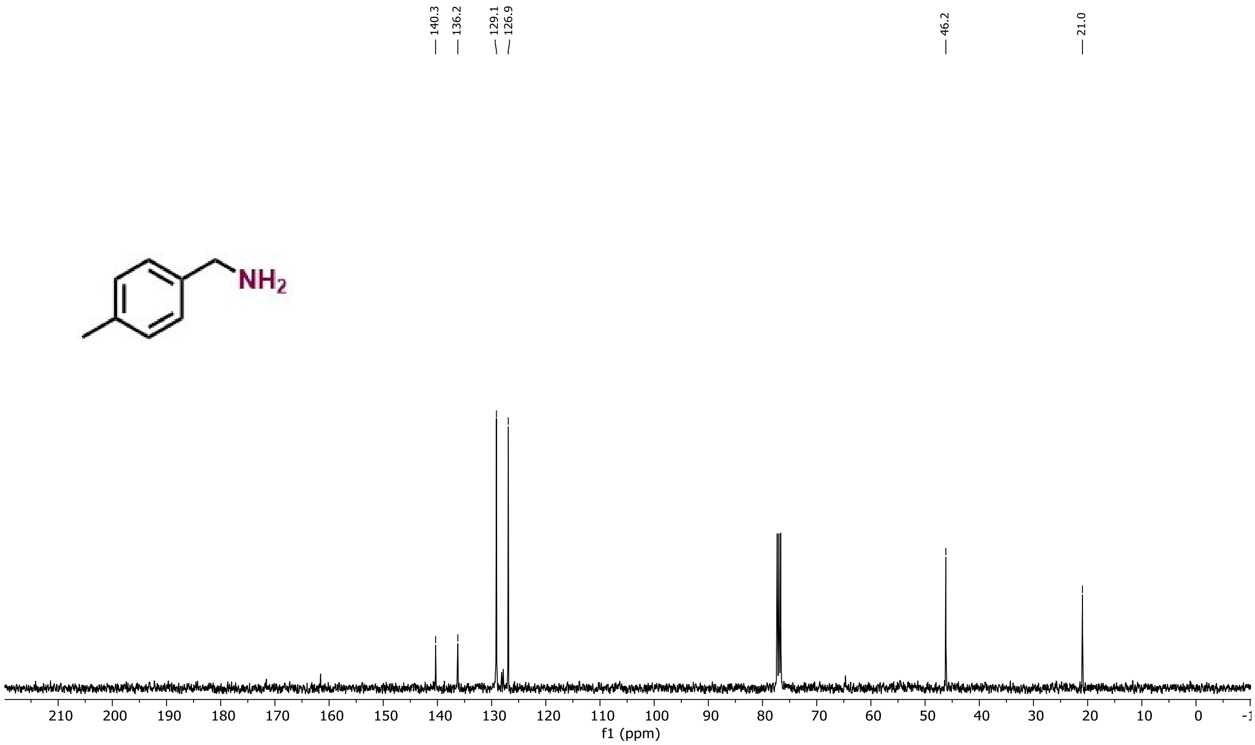

^{13}C NMR (101 MHz, $CDCl_3$) 4-Methylbenzyl amine (**3j**)

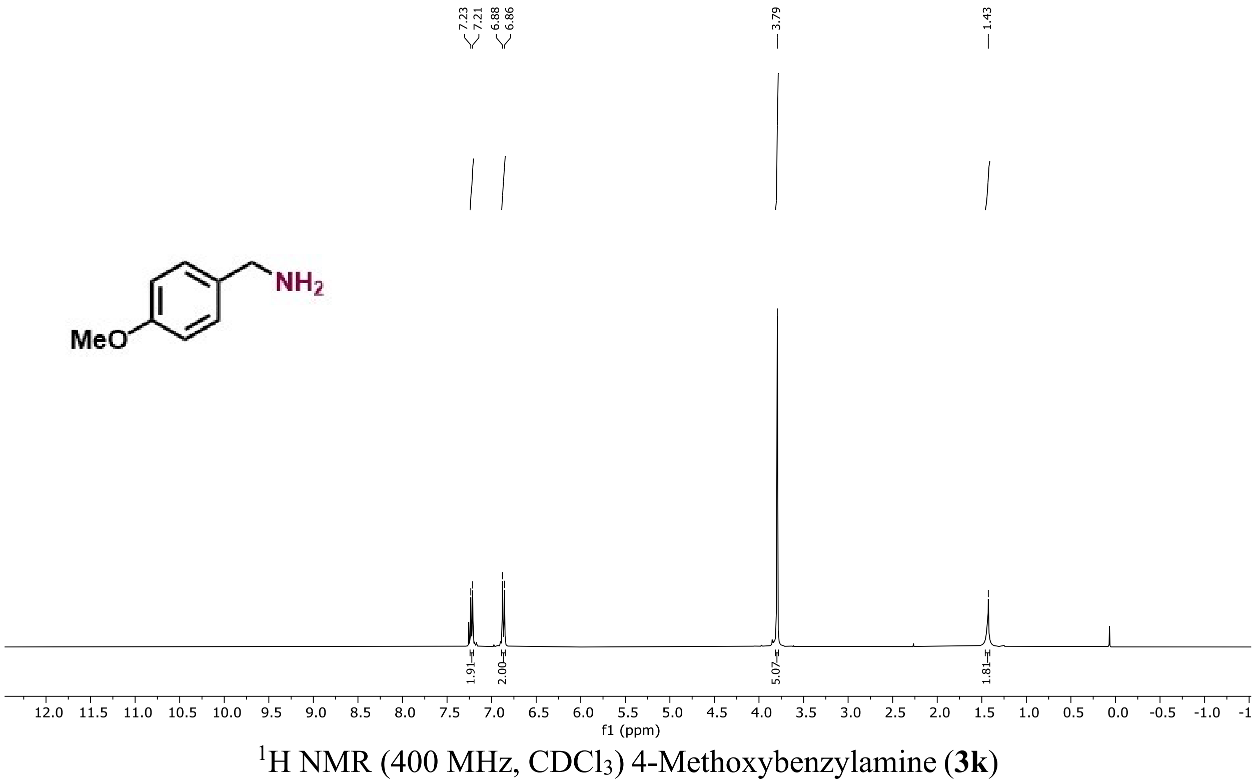

^{1}H NMR (400 MHz, $CDCl_3$) 4-Methoxybenzylamine (**3k**)

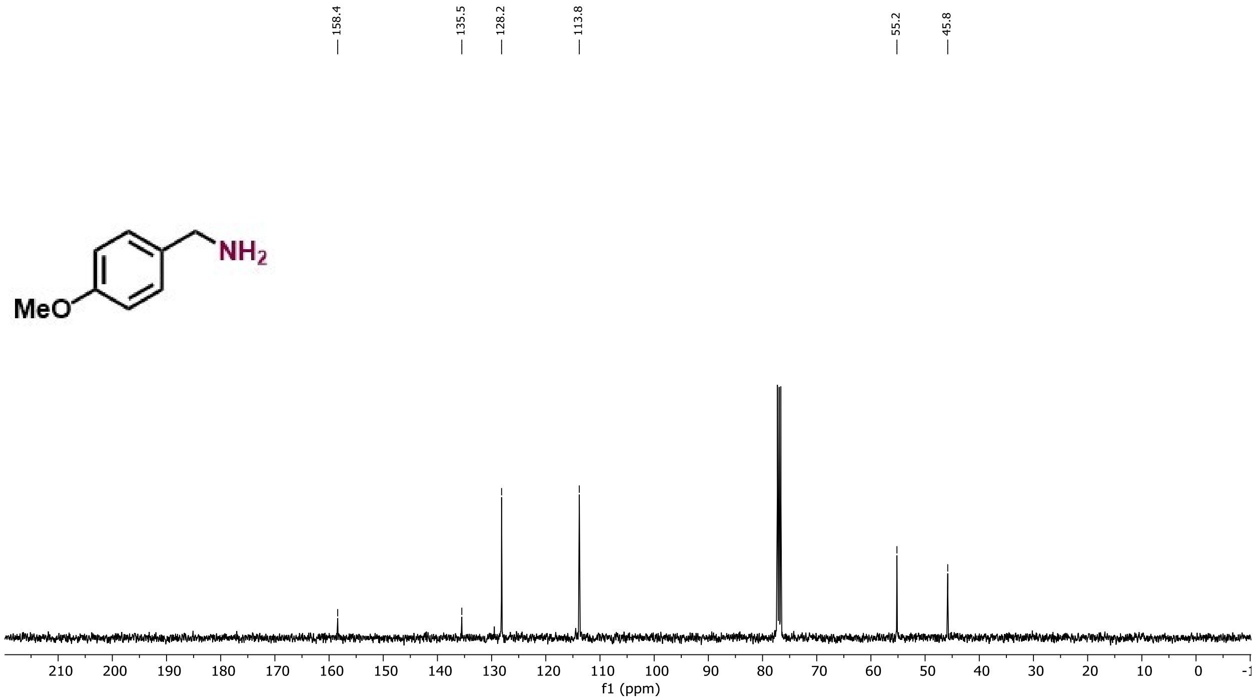

^{13}C NMR (101 MHz, $CDCl_3$) 4-Methoxybenzylamine (**3k**)

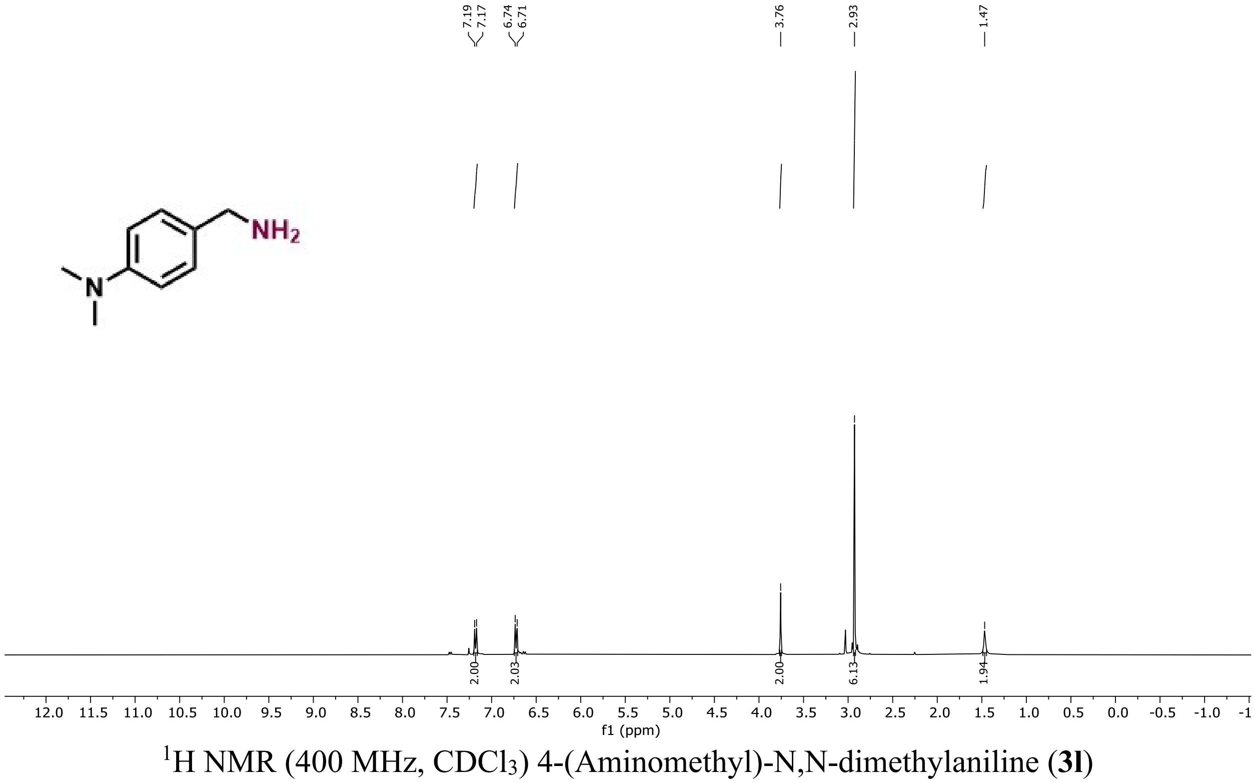

^{1}H NMR (400 MHz, $CDCl_3$) 4-(Aminomethyl)-N,N-dimethylaniline (**3l**)

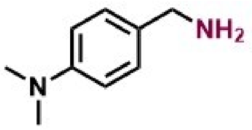

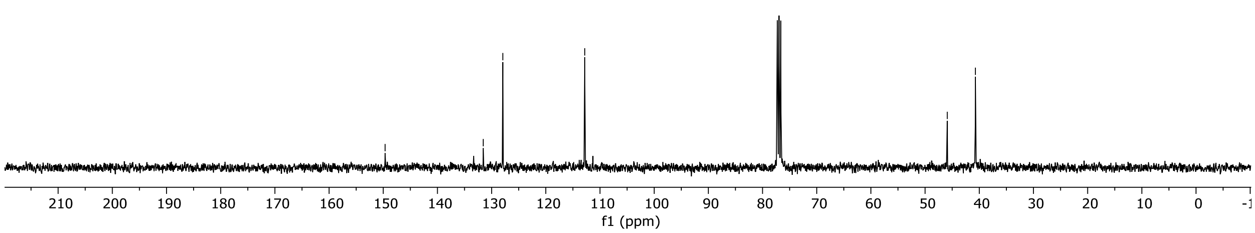

^{13}C NMR (101 MHz, $CDCl_3$) 4-(Aminomethyl)-N,N-dimethylaniline (**3l**)

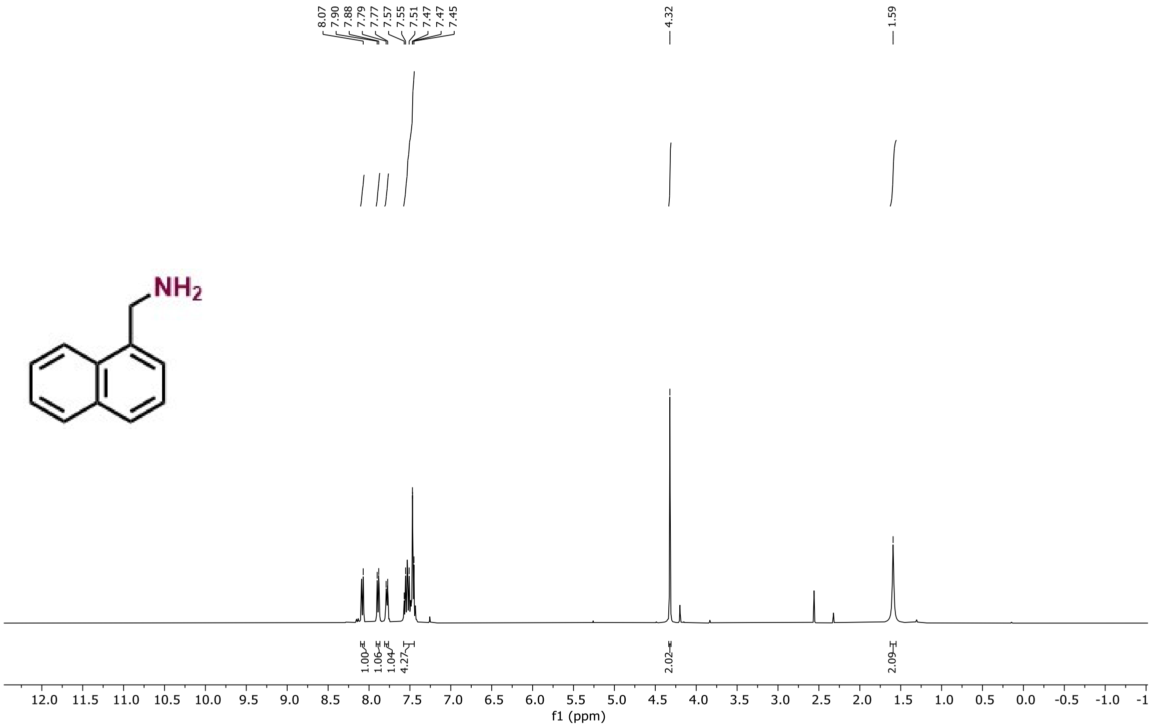

^1H NMR (400 MHz, $CDCl_3$) Naphthalen-1-ylmethanamine (**3m**)

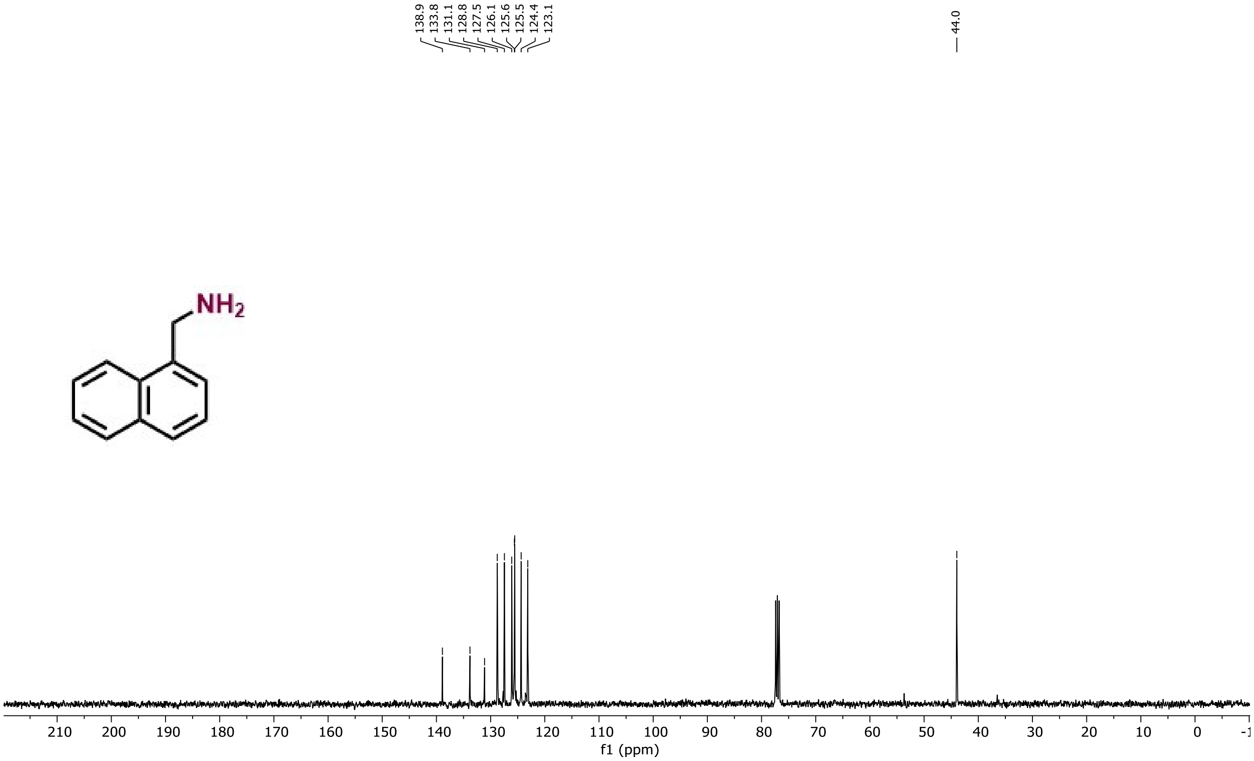

^{13}C NMR (101 MHz, $CDCl_3$) Naphthalen-1-ylmethanamine (**3m**)

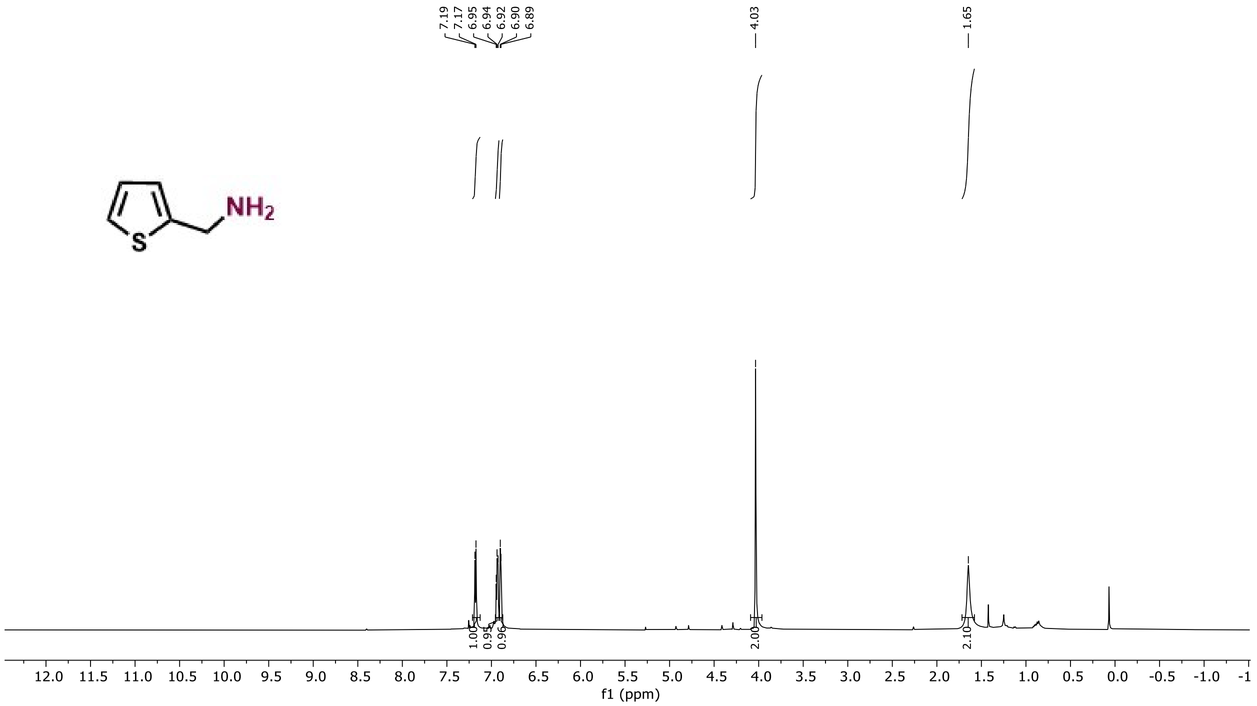

^{1}H NMR (400 MHz, $CDCl_3$) Thiophen-2-ylmethanamine (**3n**)

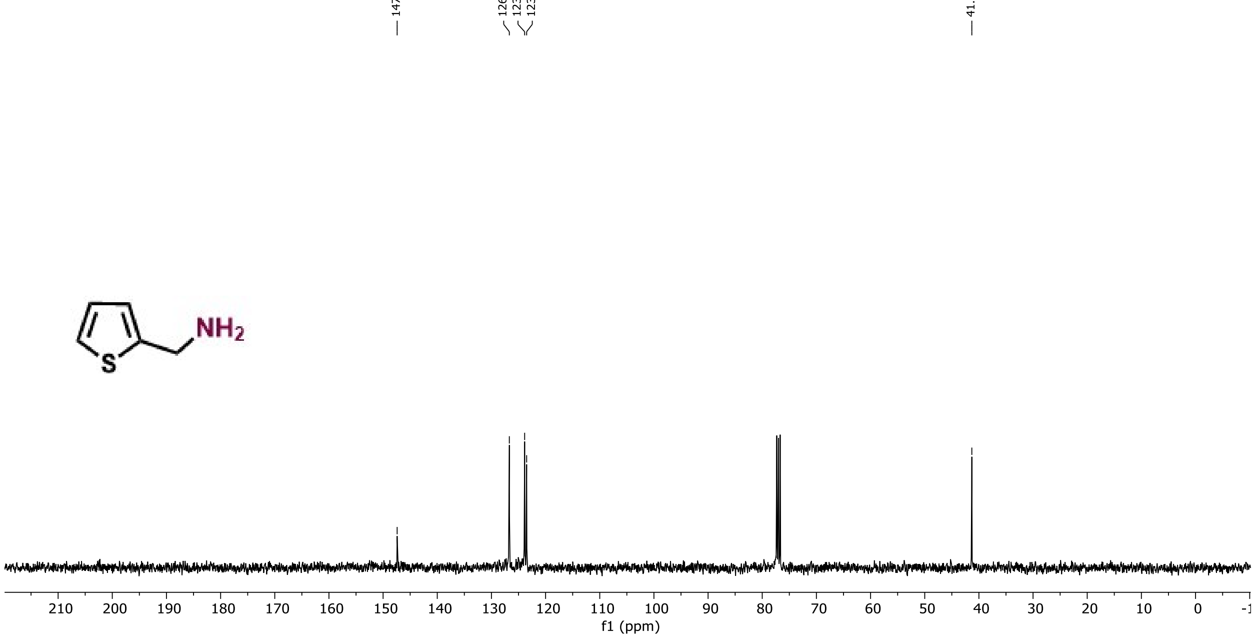

^{13}C NMR (101 MHz, $CDCl_3$) Thiophen-2-ylmethanamine (**3n**)

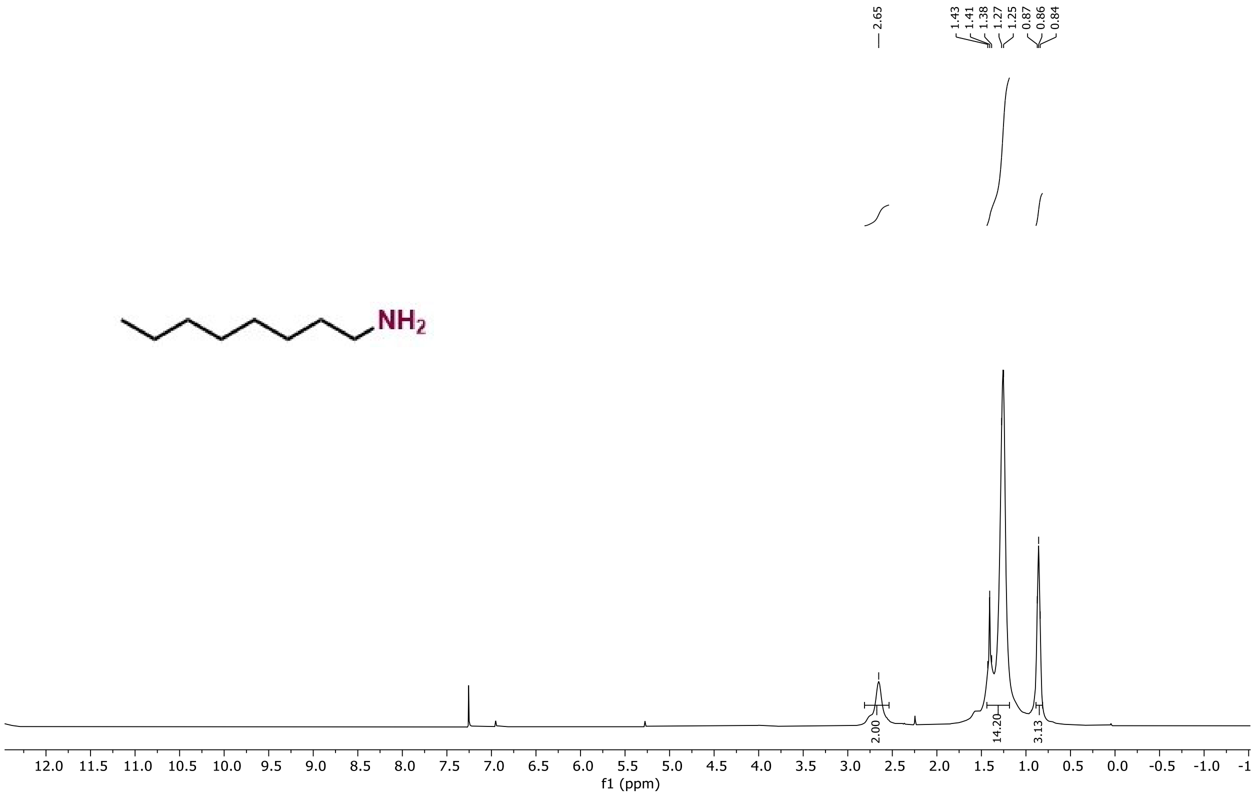

^{1}H NMR (400 MHz, $CDCl_3$) Octan-1-amine (**3o**)

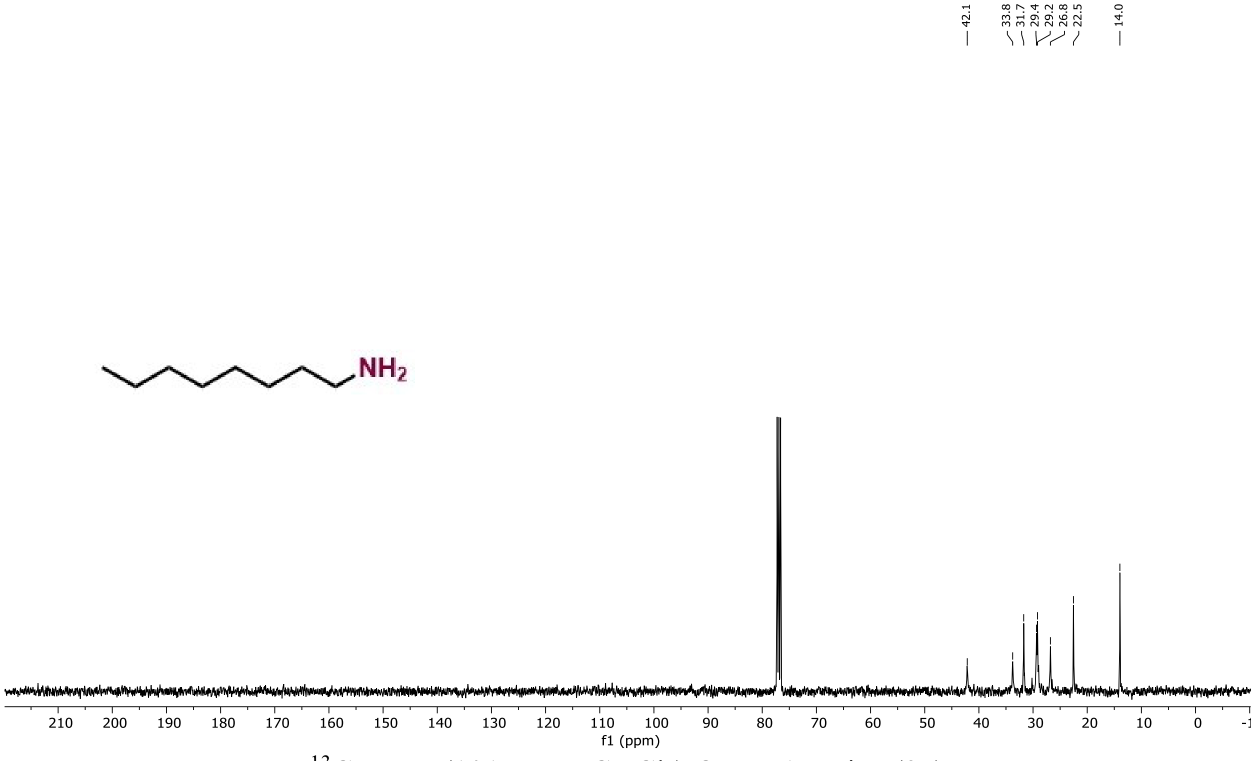

^{13}C NMR (101 MHz, $CDCl_3$) Octan-1-amine (**3o**)

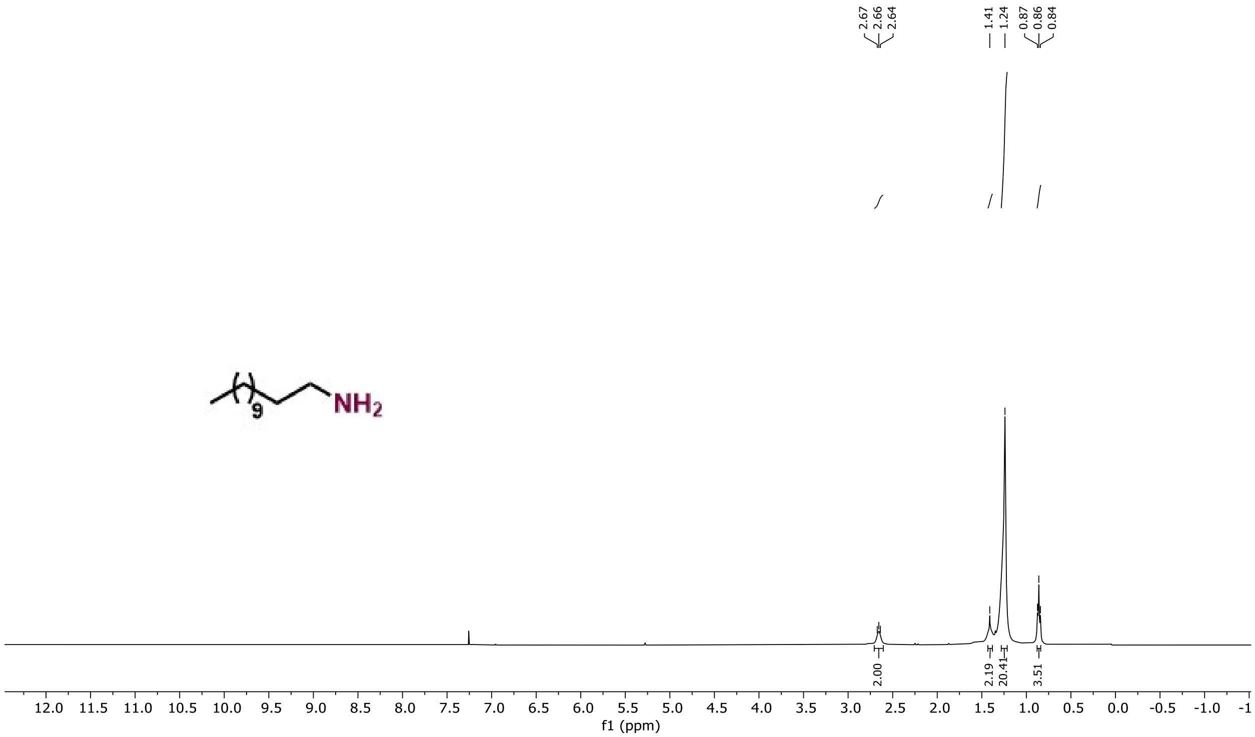

^{1}H NMR (400 MHz, $CDCl_3$) Dodecan-1-amine (**3p**)

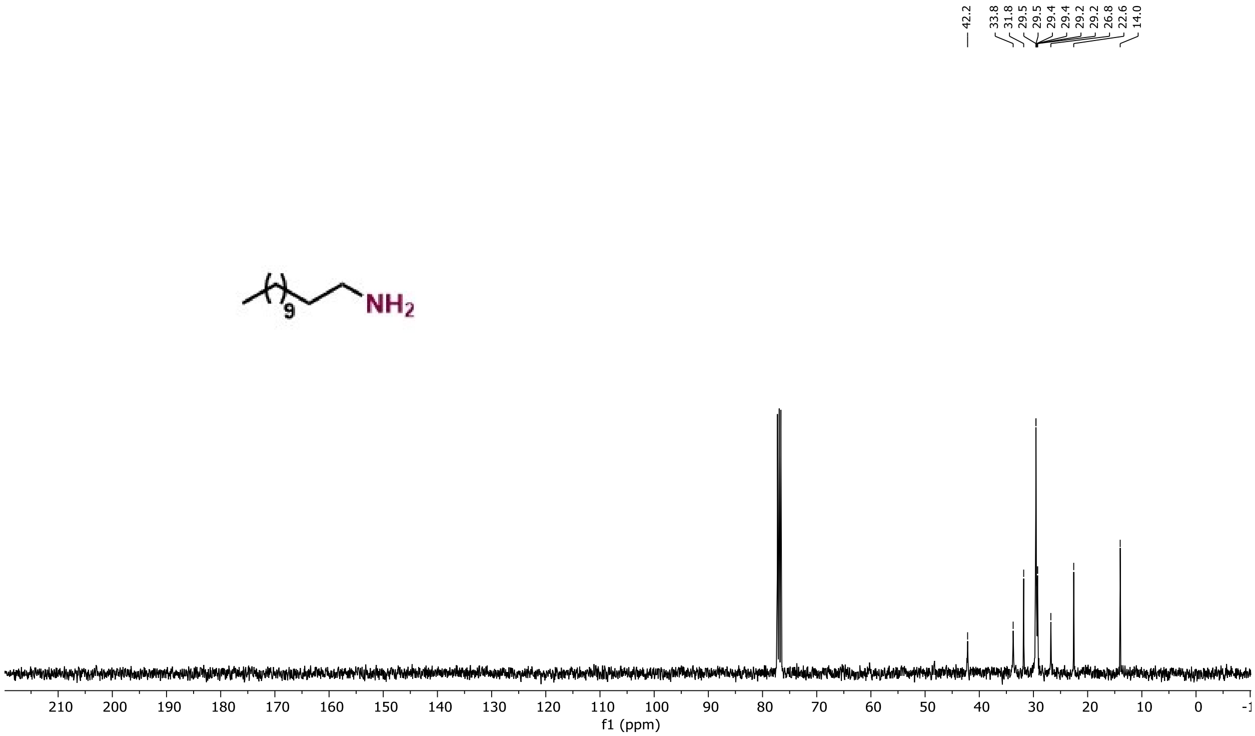

^{13}C NMR (101 MHz, $CDCl_3$) Dodecan-1-amine (**3p**)

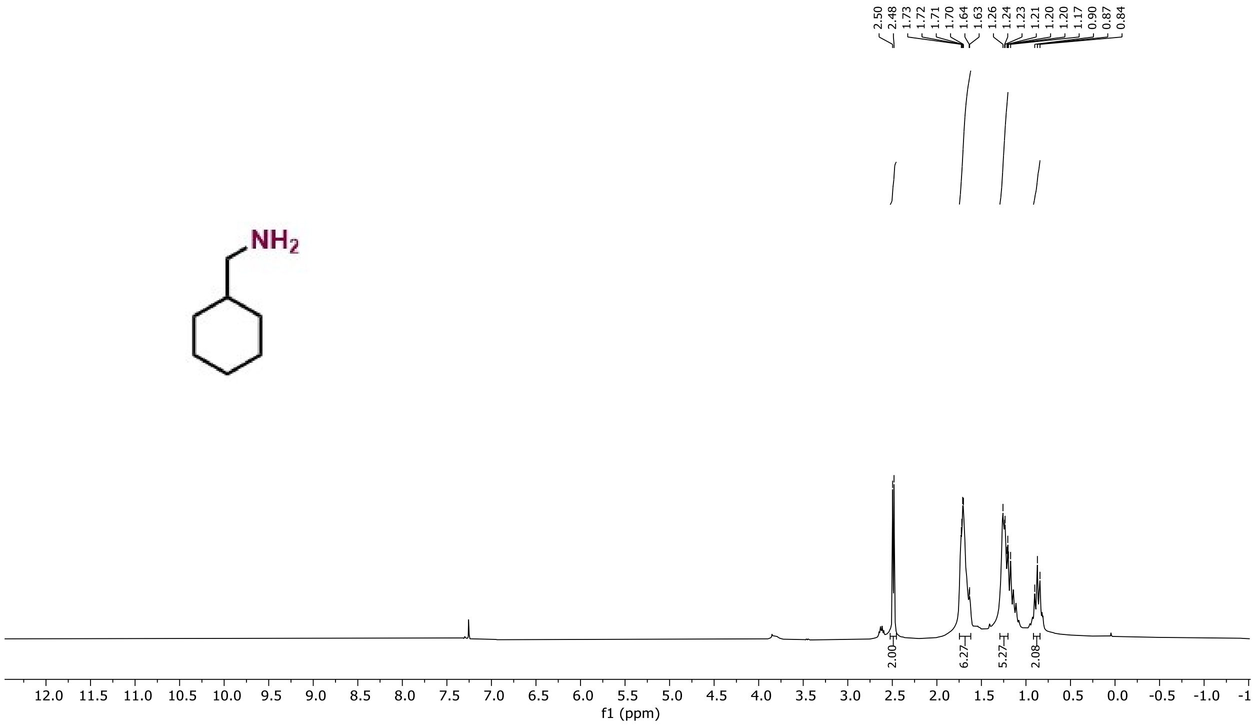

^{1}H NMR (400 MHz, $CDCl_3$) Cyclohexylmethanamine (**3q**)

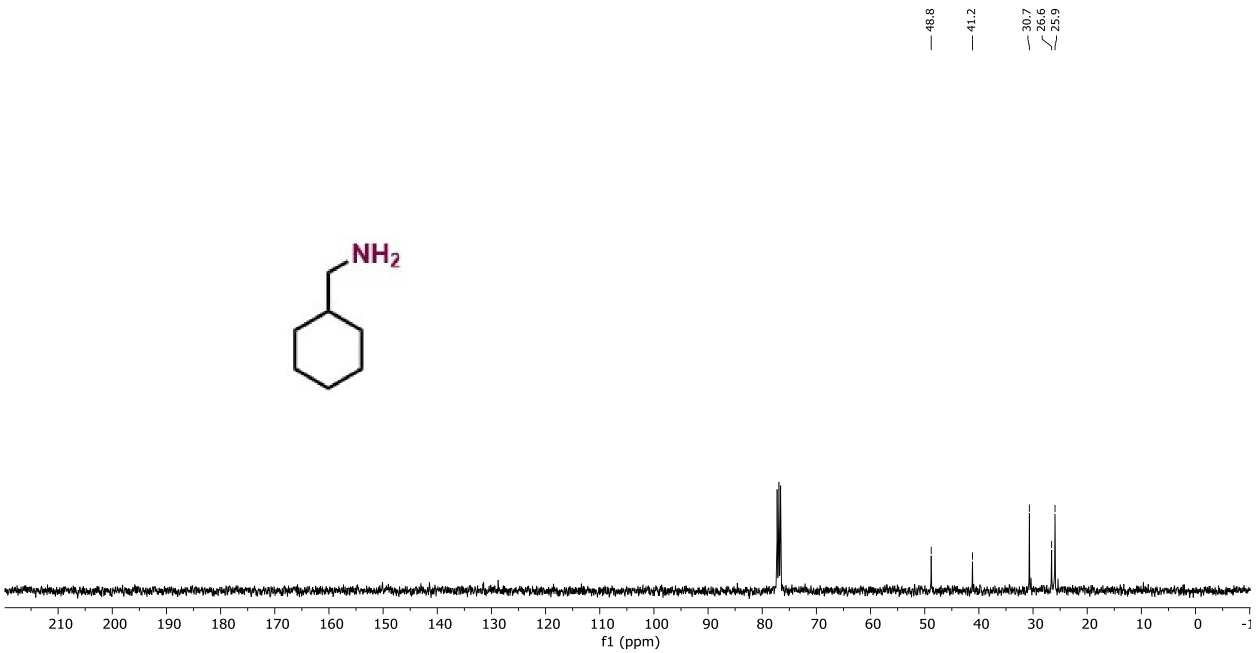

^{13}C NMR (101 MHz, $CDCl_3$) Cyclohexylmethanamine (**3q**)

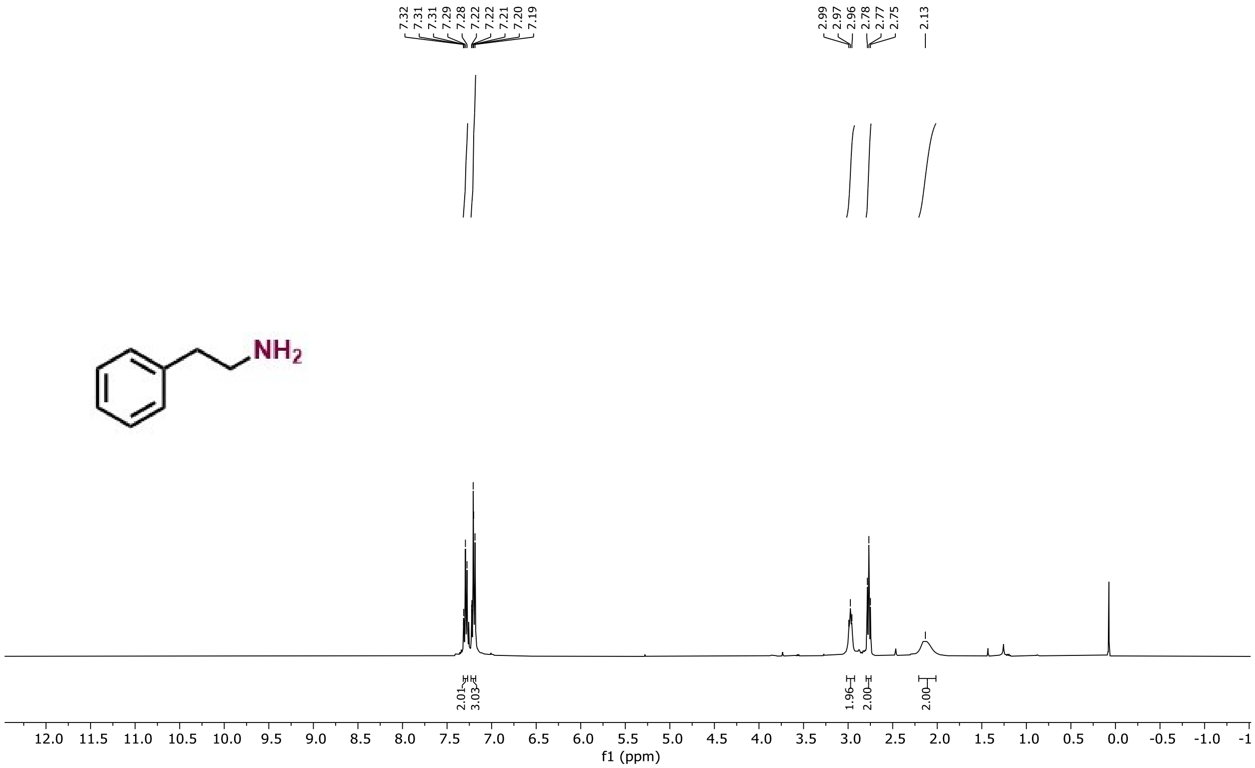

^{1}H NMR (400 MHz, $CDCl_3$) Phenethylamine (**3r**)

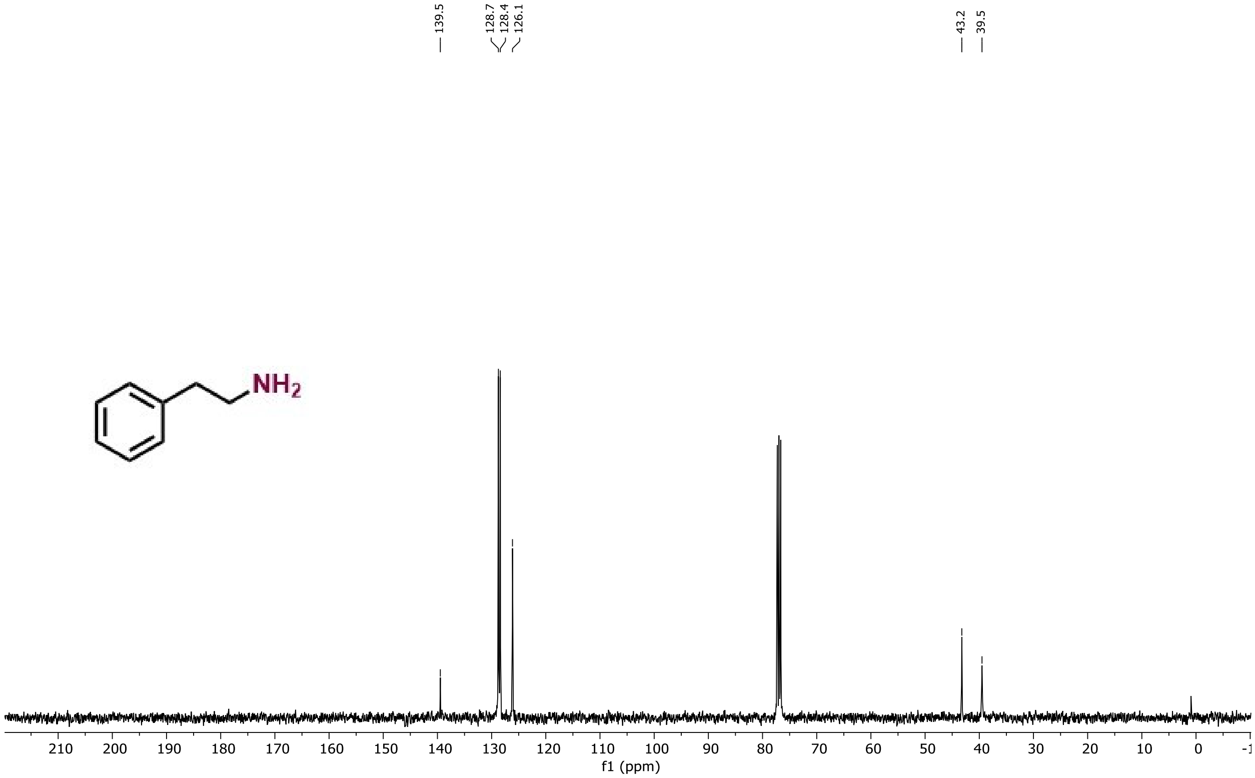

^{13}C NMR (101 MHz, $CDCl_3$) Phenethylamine (**3r**)

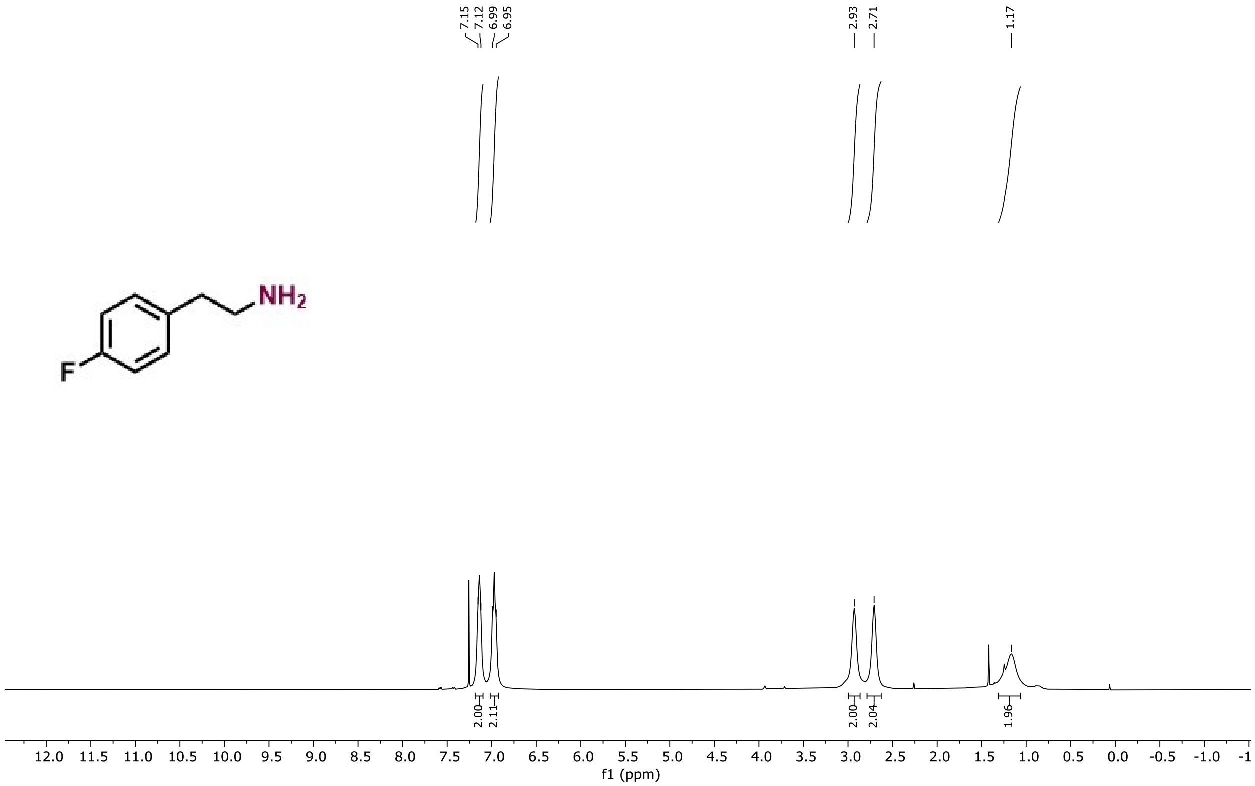

^{1}H NMR (400 MHz, $CDCl_3$) 2-(4-Fluorophenyl)ethan-1-amine (**3s**)

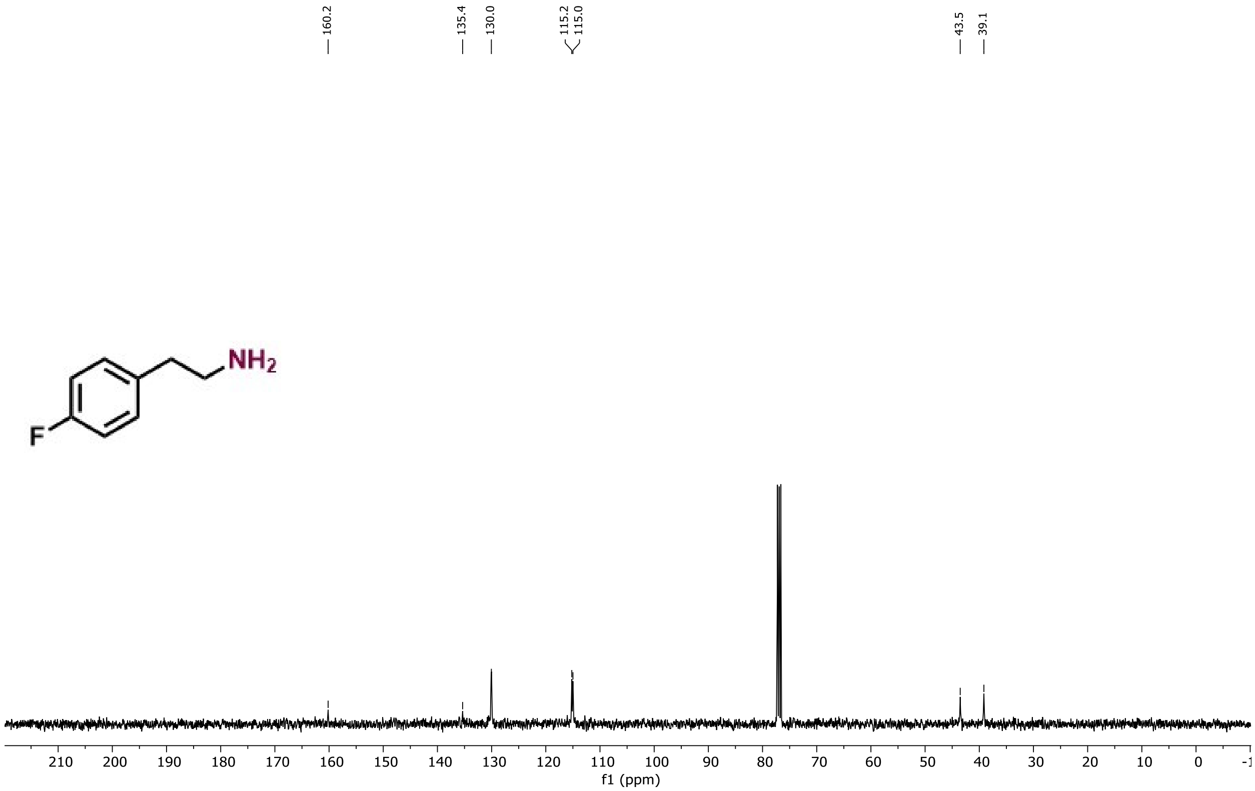

^{13}C NMR (101 MHz, $CDCl_3$) 2-(4-Fluorophenyl)ethan-1-amine (**3s**)

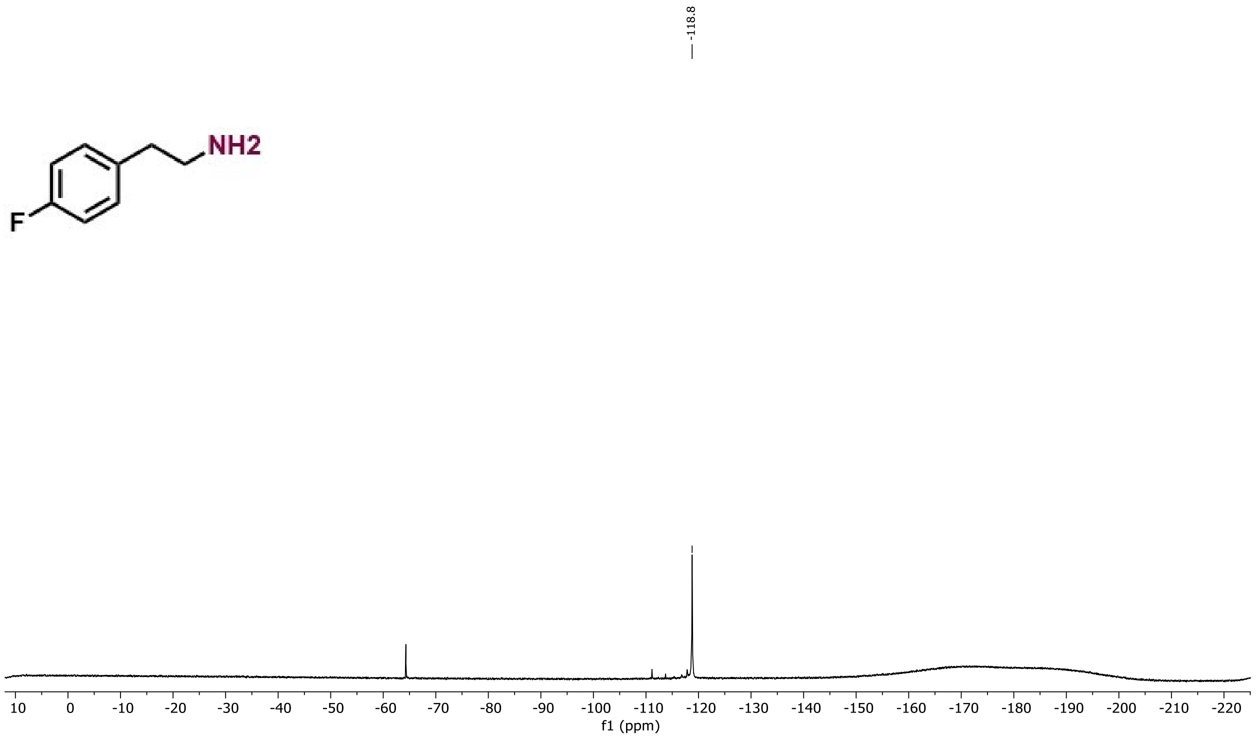

^{19}F NMR (282 MHz, $CDCl_3$) 2-(4-Fluorophenyl)ethan-1-amine (**3s**)

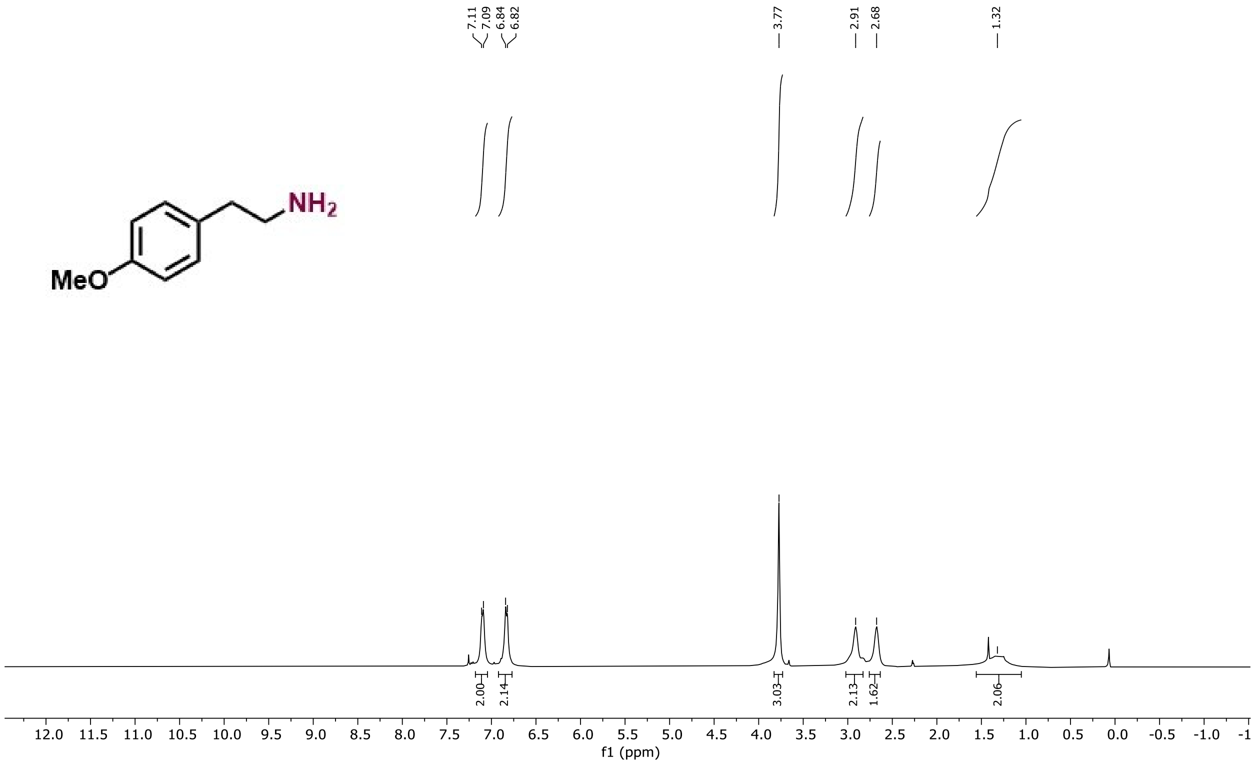

^{1}H NMR (400 MHz, $CDCl_3$) 2-(4-Methoxyphenyl)ethanamine (**3t**)

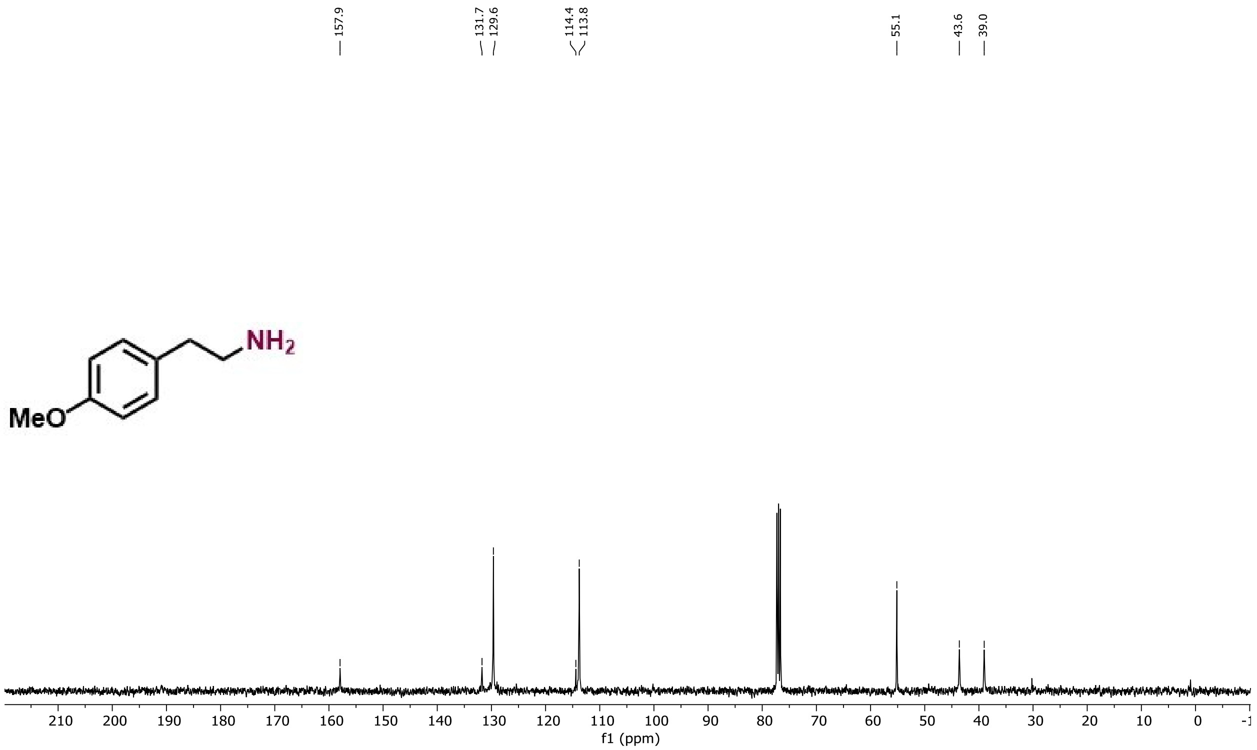

^{13}C NMR (101 MHz, $CDCl_3$) 2-(4-Methoxyphenyl)ethanamine (**3t**)

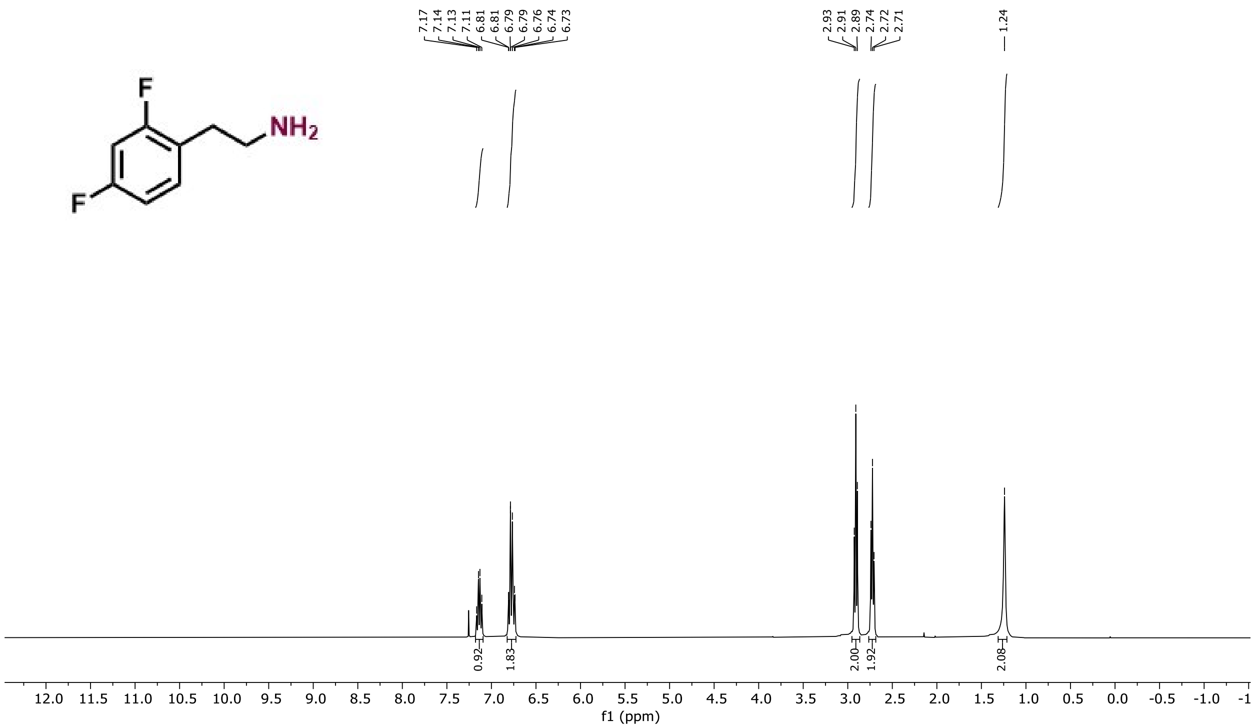

^{1}H NMR (400 MHz, $CDCl_3$) 2-(2,4-difluorophenyl)ethan-1-amine (**3u**)

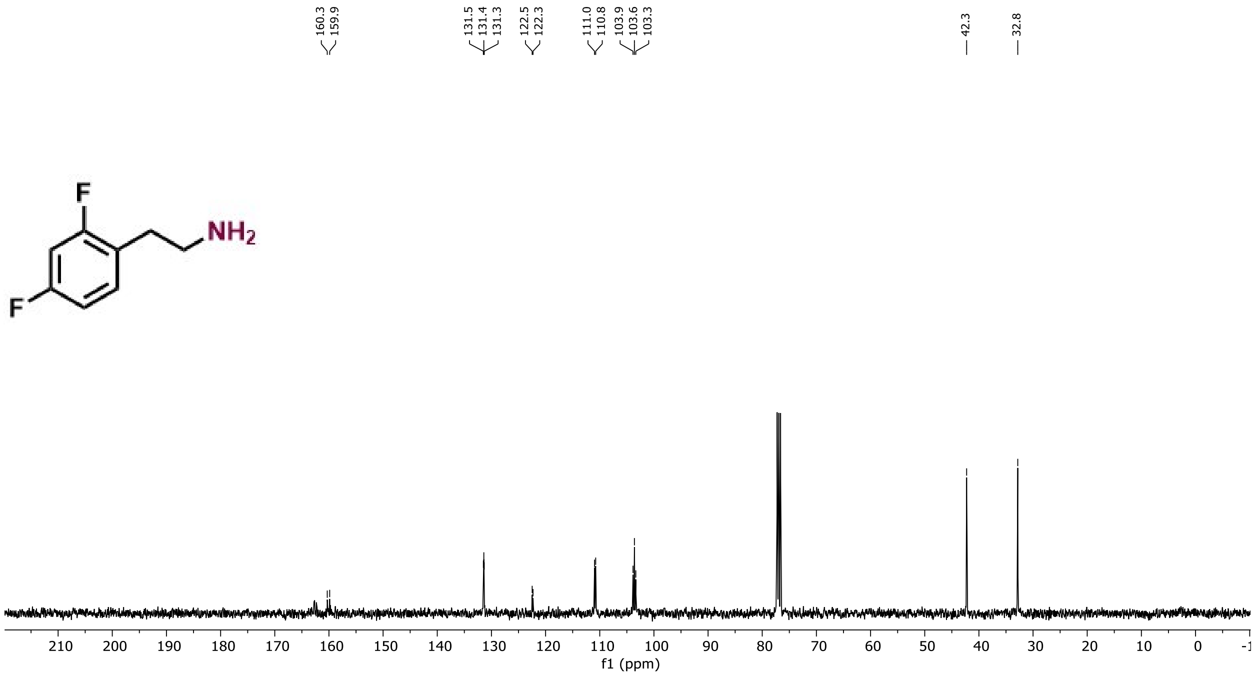

^{13}C NMR (101 MHz, $CDCl_3$) 2-(2,4-difluorophenyl)ethan-1-amine (**3u**)

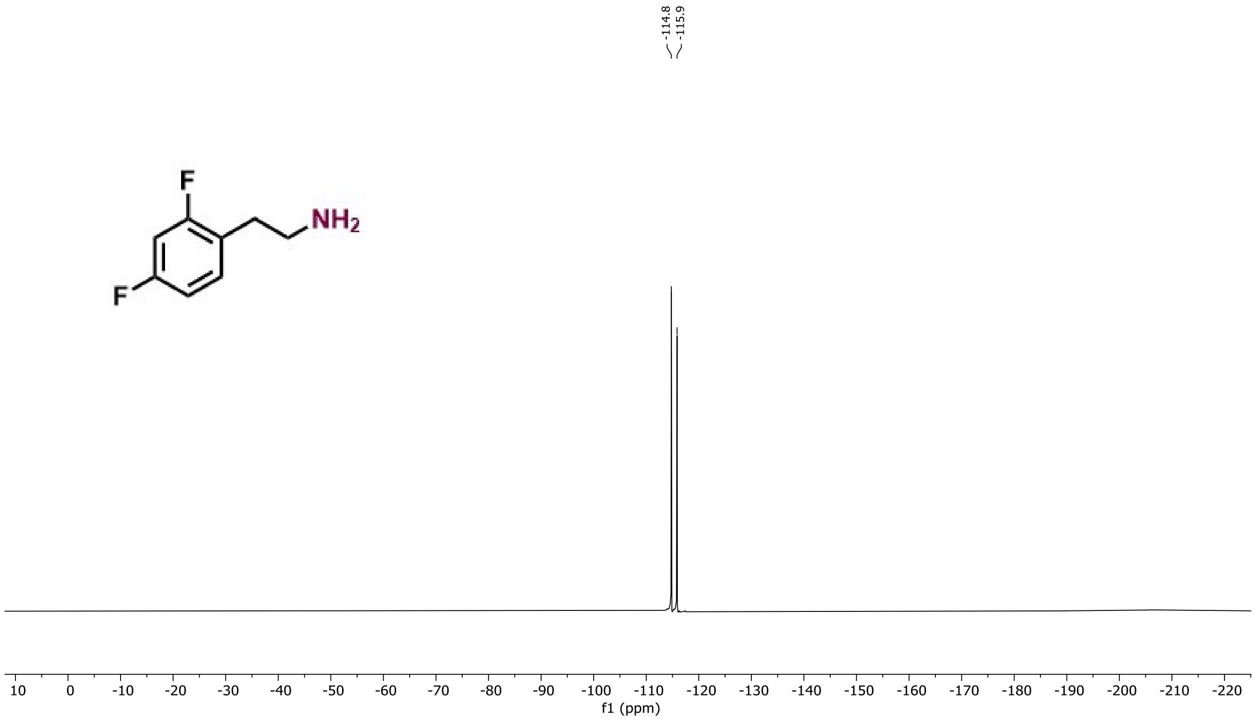

^{19}F NMR (282 MHz, $CDCl_3$) 2-(2,4-difluorophenyl)ethan-1-amine (**3u**)

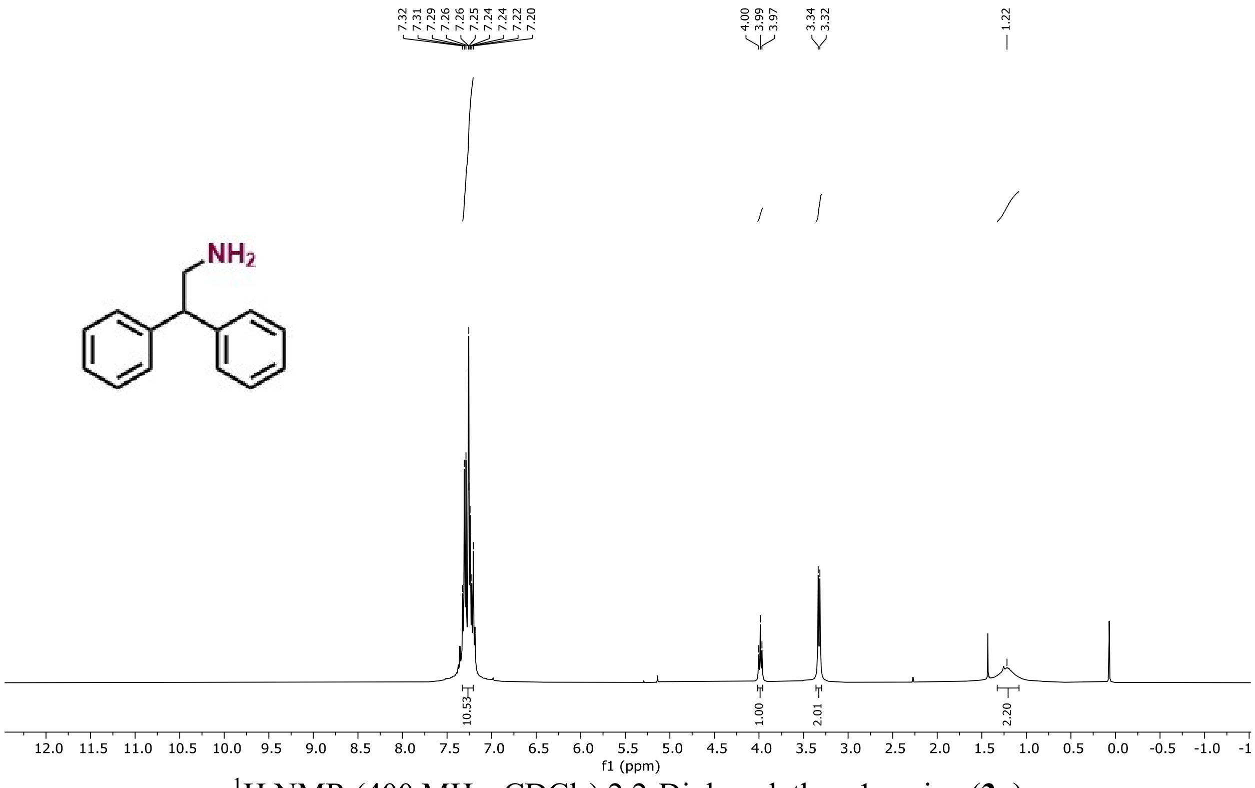

^{1}H NMR (400 MHz, $CDCl_3$) 2,2-Diphenylethan-1-amine (**3v**)

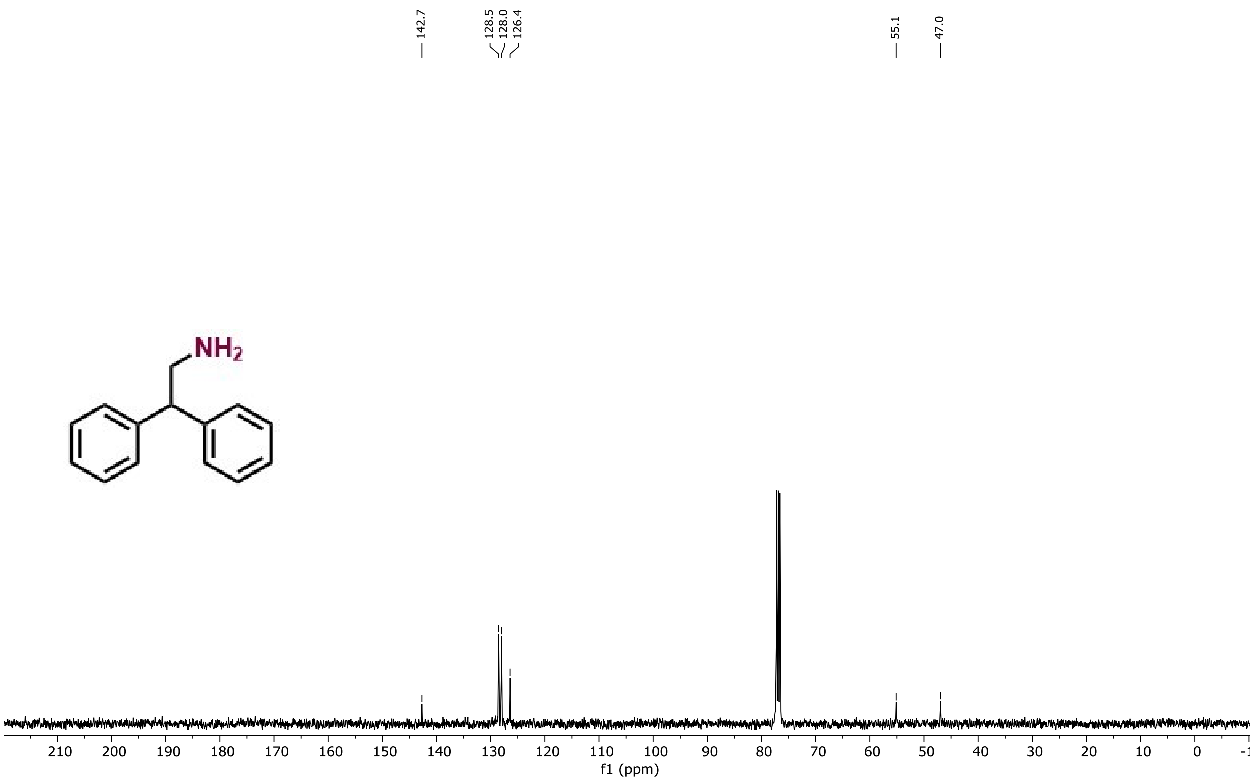

^{13}C NMR (101 MHz, $CDCl_3$) 2,2-Diphenylethan-1-amine (**3v**)
